# An Endophytic Bacterial Consortium modulates multiple strategies to improve Arsenic Phytoremediation Efficacy in *Solanum nigrum*

**DOI:** 10.1038/s41598-018-25306-x

**Published:** 2018-05-03

**Authors:** Gairik Mukherjee, Chinmay Saha, Nabanita Naskar, Abhishek Mukherjee, Arghya Mukherjee, Susanta Lahiri, Arun Lahiri Majumder, Anindita Seal

**Affiliations:** 1 Department of Biotechnology, Dr. B. C. Guha Centre for Genetic Engineering and Biotechnology, University of Calcutta, 35, Ballygunge Circular Road, Kolkata, 700019 India; 2 Department of Endocrinology & Metabolism, Institute Of Post Graduate Medical Education & Research and SSKM Hospital, Room No. 9A, 4th Floor, Ronald Ross Building, 244, AJC Bose Road, Kolkata, 700020 India; 3 Department of Environmental Science, University of Calcutta, 35, Ballygunge Circular Road, Kolkata, 700019 India; 4 Saha Institute of Nuclear Physics, Sector - 1, Block - AF Bidhannagar, Kolkata, 700064 India; 5 Homi Bhabha National Institute, 1/AF Bidhannagar, Kolkata, 700064 India; 6 Division of Plant Biology, Bose Institute, P-1/12 CIT Scheme VIIM, Kolkata, 700054 India

## Abstract

Endophytic microbes isolated from plants growing in contaminated habitats possess specialized properties that help their host detoxify the contaminant/s. The possibility of using microbe-assisted phytoremediation for the clean-up of Arsenic (As) contaminated soils of the Ganga-Brahmaputra delta of India, was explored using As-tolerant endophytic microbes from an As-tolerant plant *Lantana camara* collected from the contaminated site and an intermediate As-accumulator plant *Solanum nigrum*. Endophytes from *L*. *camara* established within *S*. *nigrum* as a surrogate host. The microbes most effectively improved plant growth besides increasing bioaccumulation and root-to-shoot transport of As when applied as a consortium. Better phosphate nutrition, photosynthetic performance, and elevated glutathione levels were observed in consortium-treated plants particularly under As-stress. The consortium maintained heightened ROS levels in the plant without any deleterious effect and concomitantly boosted distinct antioxidant defense mechanisms in the shoot and root of As-treated plants. Increased consortium-mediated As(V) to As(III) conversion appeared to be a crucial step in As-detoxification/translocation. Four aquaporins were differentially regulated by the endophytes and/or As. The most interesting finding was the strong upregulation of an MRP transporter in the root by the As + endophytes, which suggested a major alteration of As-detoxification/accumulation pattern upon endophyte treatment that improved As-phytoremediation.

## Introduction

Arsenic (As) contamination in groundwater is a major health hazard in widely distributed areas of Eastern India (West Bengal) and Bangladesh^1^ where the As-levels in potable water exceed the limit approved (10 ppb) by World Health Organization (WHO)^2^. Arsenic content in soil increases from constant irrigation with contaminated groundwater or through anthropogenic activities^3^; wherefrom it accumulates in food crops and eventually enters the food chain^4^. Even though As in drinking water is considered as the major cause of As-related diseases, excess As-content in the soil cannot also be overlooked. Although Arsenicosis is widespread among the residents of West Bengal and Bangladesh, contamination levels in the soil in these areas appear to be often ≤25 ppm. Such soil is graded as ‘low’ As-contaminated soil^1^. Highly contaminated areas in the world contain >500 ppm of As like the Zarshuran mining area in Iran where the contamination levels reach 6000 ppm. However, these areas are sparsely populated in contrast to the highly populated areas of the Gangetic delta. This makes the development of suitable strategies for clean-up of low contaminated areas extremely important. Metal contamination in soil is particularly difficult and extremely expensive to remove. The use of plants and their associated microbes to remediate contaminated soil, called phytoremediation, is an extremely relevant strategy in this context.

Plant and its associated microbes participate in complex interactions that shape the community structure of these microbes and also the plant’s interaction with its environment. Endophytes are microbes that reside asymptomatically within the plant endosphere. Properties of the endophytes are dictated by the edaphic factors wherein the host plant survives. While one of the selection forces determining the endophytic colonization is the nutritional status of the host^5^, the necessity to detoxify a xenobiotic appears to be an additional driving force. This is exemplified by the fact that the endophytic microbes isolated from plants surviving in contaminated habitats often contain properties useful for the detoxification of the related xenobiotic/s^6^. Endophytes isolated from plants growing in metal-contaminated habitats have been reported to harbor genome or plasmid coded strategies to detoxify metals^7^ and contribute to the metal tolerance and accumulation property of the host plant. An endophyte related to *Achromobacter piechaudii* from the metallophyte *Sedum plumbizincicola* demonstrated increased uptake of different metals in the root^8^. *Enterobacter* sp. PDN3, from poplar growing in areas polluted with organic xenobiotics, was able to degrade trichloroethylene (TCE)^6^. Another *Enterobacter* sp. from water hyacinth contained a plasmid responsible for conferring metal tolerance to the bacteria.

Endophytes exert their plant growth promoting (PGP) properties on their hosts in a way analogous to the gut microflora of animals^9^. Although host genotype impacts their properties to a certain degree, a number of studies have shown that this influence is limited indicating that the soil condition (also determining the plant’s nutritional status) predominately drives their entry into a plant^10,11^. Endophytes from *Pteris vittata*- an As hyperaccumulator were found to transform As(V) to As(III) and produced IAA, solubilized phosphate, and siderophores as mechanisms of growth promotion^12^. In contrast, endophytes isolated from *S*. *nigrum* growing at a mining site contaminated with different heavy metals were mostly siderophore and ACC deaminase producing^13^. Recent studies have indicated that endophytes can also have beneficial effects on distantly related plant genera besides their natural host^14,15^. This is extremely significant because this opens up the possibility that the endophytes can be used to transmit their properties to surrogate plants of agricultural value and also to plants relevant for phytoremediation. For example, nitrogen-fixing endophytes isolated from *Typha angustifolia* collected from a nutrient-deficient Uranium mine improved nitrogen metabolism in rice^16^.

We examined the possibility of using microbe-assisted phytoremediation in the clean-up of soil containing As-contamination levels relevant to highly populated areas of Gangetic West Bengal. As-resistant microbes were isolated from an As-tolerant excluder *Lantana camara* collected from a contaminated site in Nadia, West Bengal^17^ and was applied to a perennial weed *Solanum nigrum* found in abundance in this area for the purpose of As-phytoremediation. *S*. *nigrum* is an established Cadmium (Cd)-hyperaccumulator that has been reported to be an intermediate As-accumulator able to store up to 500 ppm of As^18^. A significant amount of this As is accumulated in the shoot^19^ making this plant suitable for As-phytoremediation^18^. Ferns of genus *Pteris* especially *Pteris vittata*^20^ are widely studied for their unique ability to accumulate over 4000 ppm of As in the above-ground biomass. However, the rhizome and rhizoids of these ferns penetrate only the surface layers of soil restricting their use in *in situ* phytoremediation. Use of hyperaccumulators in soil clean-up is often limited by their slow and stunted growth. Higher biomass increases the effectiveness at which a soil pollutant can be removed. *Brassica juncea*^21,22^ and *Brassica carinata* are reported to accumulate As^23^. Although not hyperaccumulators these are fast growing plants. However, their leaves and seeds are widely consumed making them undesirable as phytoremediation models. Naser Karimi and his colleagues (2009) reported *Isatis cappadocica*^24^ and *Hesperis persica*^25^ as As-accumulator terrestrial angiosperms. Although both these plants are highly tolerant to As, the bioaccumulation factor reported (shoot: soil concentration) was low; approximately 1 and 0.89 respectively^24,25^. Further, *Isatis sp*. and *Hesperis* sp. are not endemic to As-contaminated regions of the Gangetic plains of India neither has their As-accumulation ability been tested at low concentrations of As. This prompted us to search for suitable local plant/s. *S*. *nigrum* appeared to be a suitable plant host for microbe-assisted As-phytoremediation of low As-contaminated soil. It is a weed well adapted to the As-contaminated region under study showing a much wider geographic distribution.

The As-tolerant endophytic consortium from *L*. *camara* was found to improve the As-phytoremediation efficiency of *S*. *nigrum* without the necessity of a transgenic approach, influencing multiple processes known from previous studies to improve As-tolerance. While individually, the microbes had a variable effect, some showing growth promotion but having little or no role in the improvement of As-bioaccumulation or vice versa, when used as a consortium, they efficiently improved As-accumulation in *S*. *nigrum*. Our work demonstrated the suitability of using endophytes possessing desired properties coupled with an appropriate plant model in As-phytoremediation even when the natural host of the microbes was unsuitable for the application.

## Results and Discussions

### Solanum nigrum - a suitable model for microbe-assisted Arsenic remediation

To identify an As-accumulator terrestrial plant (i) a non-biased, as well as (ii) a targeted approach was taken. For the non-biased approach, non-edible plants with As-bioaccumulation factor (shoot/rhizospheric soil As concentration) >1 were searched from an As-contaminated site in Nadia, previously reported for groundwater contamination and As-related diseases. The concentration of As in plant rhizosphere measured by VGA-AAS was found to be 19.3(±3.77) ppm. None of the plants from 14 different species studied from the site were As-accumulators (data not shown). This was not surprising as the exclusion of metals is a predominant strategy adopted by many metallicolous plants adapting to contaminated soils^26^. *Lantana camara -*a perennial weed was one of the highest accumulators of As among the collected plants, [12.5(±0.25) ppm in shoot] and was therefore selected for isolation of As-tolerant endophytes. Arsenic was supplied as arsenate (sodium arsenate, As hereafter)− the main form of As in aerobic soil. Albeit tolerant to 25 ppm of As under laboratory conditions, *L*. *camara* showed a bioaccumulation factor <1. Metal-bioaccumulation is a property largely dictated by the plant and is regulated by complex strategies controlling uptake, root to shoot transport and detoxification of the metal. We envisaged that a successful microbe-assisted phytoremediation model would require an As-accumulator plant model.

A targeted approach was therefore adopted and *Solanum nigrum*, an already known As-accumulator plant^19,27^ was used as the plant model. We hypothesized that *S*. *nigrum*, being an As-accumulator is equipped with strategies to take up, transport and store As at high amounts- a prerequisite to As-phytoremediation. Although not found at the time of collection, *S*. *nigrum* is a non-edible short-lived perennial shrub widely found in this region. It is not an As-hyperaccumulator but has a significant biomass and a robust root system. *S*. *nigrum* (tetraploid Red Makoi variety) accumulated 152.3 (±12.4)ppm of As in its shoot under laboratory conditions when supplied with 25 ppm of As, which was the upper limit of the As found in the soil of the studied area. Although this As-level was much lower than that reported in Iran by Karimi *et al*.^24^ or in the mine tailings of Mexico^28^ the amount was still 2500-fold higher than the acceptable limits and was much more relevant to the contamination level that poses threat to the human populace. *S*. *nigrum* has been earlier reported to restrict root to shoot translocation of As^18,19^. We found that although the As-concentration in root was higher compared to shoot, the bioaccumulation factor was still >1. *S*. *nigrum* was therefore chosen as the As-accumulator plant model in this study.

### Arsenic tolerant endophytes from *Lantana camara* contained plant growth promoting properties

**Figure 1:**
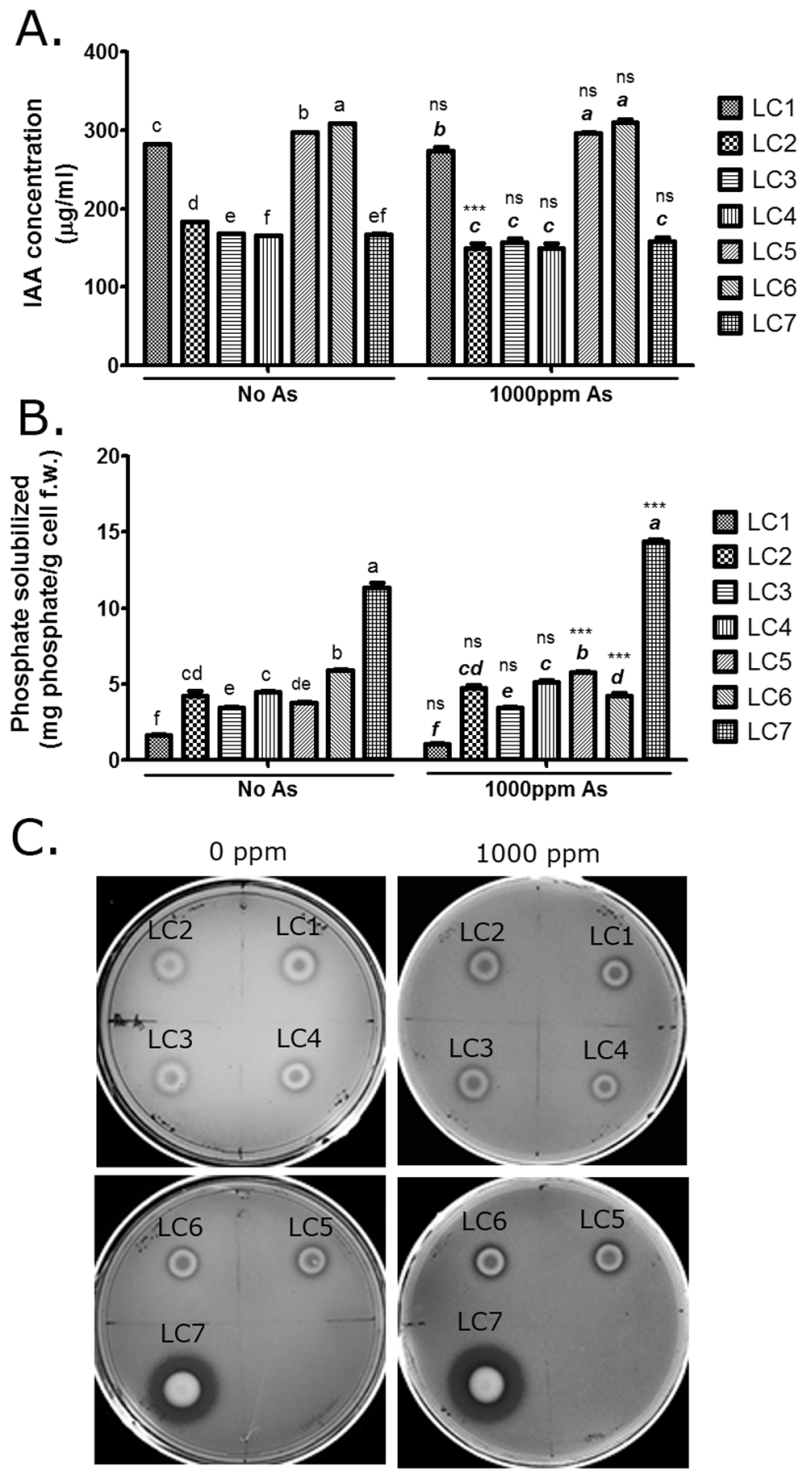
*Lantana camara* endophytes were auxin producing and phosphate solubilizing. (**A**) The endophytes were grown in Luria- tryptophan broth in the presence of 0 and 1000 ppm As. Auxin production was quantified in culture supernatants at A_540_ against a standard curve of IAA; n = 18 (Df for As:1, for endophytes:6, for interaction:6, error:238). (**B**) & (**C**) The endophytes were grown in Pikovskaya media in the presence of 0 and 1000 ppm As and phosphate solubilization potential was measured both quantitatively at A_880_ against a standard curve of K_2_HPO_4_; n = 12 (Df for As:2, for endophytes:6, for interaction:12, error:105), (**B**) and qualitatively by halo formation around the spotted cells (**C**). LC1.:*Enterobacter* sp. LC1, LC2.:*Kocuria* sp. LC2, LC3.:*Kocuria* sp. LC3, LC4.:*Enterobacter* sp. LC4, LC5.:*Kocuria* sp. LC5, LC6.:*Enterobacter* sp. LC6, LC7.:*Kosakonia* sp. LC7, U = Uninoculated control. Data are represented as mean ± SEM. Bars with different letters indicate significant differences amongst different endophytic isolates at a particular As-level (bold italics for +As) obtained from two-way ANOVA with Tukey’s post-hoc test. Significant differences for an individual isolate between −As and +As treatments have been marked by ***(P < 0.001); ns = no significance.

Seven bacterial endophytes from *L*. *camara* tolerant to 4000 ppm of As were isolated based on their distinct colony morphology (Fig. S1A). The 16S rDNA sequences were compared against EZBiocloud and NCBI database and the generic nomenclature was assigned according to EZBiocloud database (Table 1). Their evolutionary relationship was ascertained by a phylogenetic tree (Fig. S2). Three out of seven isolates were of *Kocuria* sp (LC2, LC3, and LC5). The rest belonged to Enterobacteriaceae *viz*. *Enterobacter* sp. (LC1, LC4, and LC6) and *Kosakonia* sp. (LC7) (Table 1). The 16S rDNA sequences of the different isolates of *Enterobacter* and *Kocuria* showed significant variations indicating considerable diversity amongst them. *Enterobacter cloacae* is already reported as endophyte of corn^29^, pine^30^ and *Capsicum*^31^. Genome sequence of an As-resistant *E*. *cloacae* LSJC7 shows a chromosomally coded arsenic resistance (ars) operon required for detoxification of arsenate, arsenite and antimonite^32^. Recently *Kocuria arsenatis*, a novel strain of As-tolerant endophytic bacteria, was isolated from *Prosopis laegivata*- a plant collected from an As-contaminated mine tailings^28^. Notably, *K*. *arsenatis* was the closest homolog of one of our isolates, *Kocuria* sp. LC5. *Kocuria palustris* was reported as another As-tolerant bacterium containing As-detoxifying genes indicating that such mechanisms may be common in genus *Kocuria*. Ars operon has been widely studied in *E*.*coli*^33,34^. Despite such mechanistic studies of As-tolerance in bacteria, its influence on As-phytoremediation in plants has not been reported. Bacterial PGP activities could result in increased plant biomass essentially increasing storage space within the plant for the accumulated As. The ability to produce growth hormone auxin (Fig. 1A), and to solubilize inorganic phosphate (P) appeared to be predominant among the PGP activities (Fig. 1B,C) both under −As and +As conditions. Interaction effects of As and endophytes on auxin production and P-solubilization were observed [F(6,238) = 5.996 (P < 0.0001) and F(12,105) = 34.75 (P < 0.0001) respectively] suggesting that these PGP activities of the endophytes were influenced by As and that the microbes were able to use these strategies to promote plant growth under As-challenged conditions. *Enterobacter* sp. LC1, *Kocuria* sp. LC5 and *Enterobacter* sp. LC6 were among the highest auxin producers (Two-way ANOVA with Tukey’s post-hoc analysis). LC7 had the highest P-solubilization potential, [14.37(±0.32) ppm in the presence of 1000 ppm As] which was increased by As-treatment (Two-way ANOVA with Tukey’s post-hoc analysis; Fig. 1B) in agreement with the qualitative assay (Fig. 1C). All the endophytes solubilized P indicating that their colonization within *L*. *camara* was likely driven by induced P-deficiency as a consequence of As-contamination in the soil since arsenate enters plants through the opportunistic use of P-transporters^35^.

**Table 1:** Identification of arsenic-tolerant endophytes from *Lantana camara* shoot.

Endophyte name	GenBank Accession No.	Maximum similarity with (In EZBioCloud) and similarity score	Maximum similarity with (in NCBI BLAST) and similarity score	Bacterial Phylum
*Enterobacter* sp. LC1	KT873248	*Enterobacter cancerogenus* LMG 2693 (Z96078), 99.52%	*Enterobacter cloacae* S20504 (KF956588), 99.3%	Proteobacteria
*Kocuria* sp. LC2	KU821101	*Kocuria rhizophila* DSM 11926 (Y16264), 99.36%	*Kocuria rhizophila* F2 (KM577162), 99.5%	Actinobacteria
*Kocuria* sp. LC3	KT873249	*Kocuria rhizophila* DSM 11926 (Y16264), 99.72%	*Kocuria rhizophila* F2 (KM577162), 98.2%	Actinobacteria
*Enterobacter* sp. LC4	KT873250	*Enterobacter hormaechei* subsp. *oharae* DSM 16687 (CP017180), 99.38%	*Enterobacter cloacae* S20504 (KF956588), 99.5%	Proteobacteria
*Kocuria* sp. LC5	KT873251	*Kocuria arsenatis* CM1E1 (KM874399), 97.16%	*Kocuria rhizophila* F2 (KM577162), 96.0%	Actinobacteria
*Enterobacter* sp. LC6	KU051718	*Enterobacter ludwigii* EN-119 (JTLO01000001), 99.24%	*Enterobacter cloacae* 34983 (CP010377), 99%	Proteobacteria
*Kosakonia* sp. LC7	KT873252	*Kosakonia cowanii* JCM 10956 (BBEU01000098), 97.91%	*Escherichia* sp. CZBRD4 (KJ184949), 97.6%	Proteobacteria

### *Lantana camara* endophytes colonized *Solanum nigrum* endosphere and promoted its growth in the presence of arsenic

**Figure 2:**
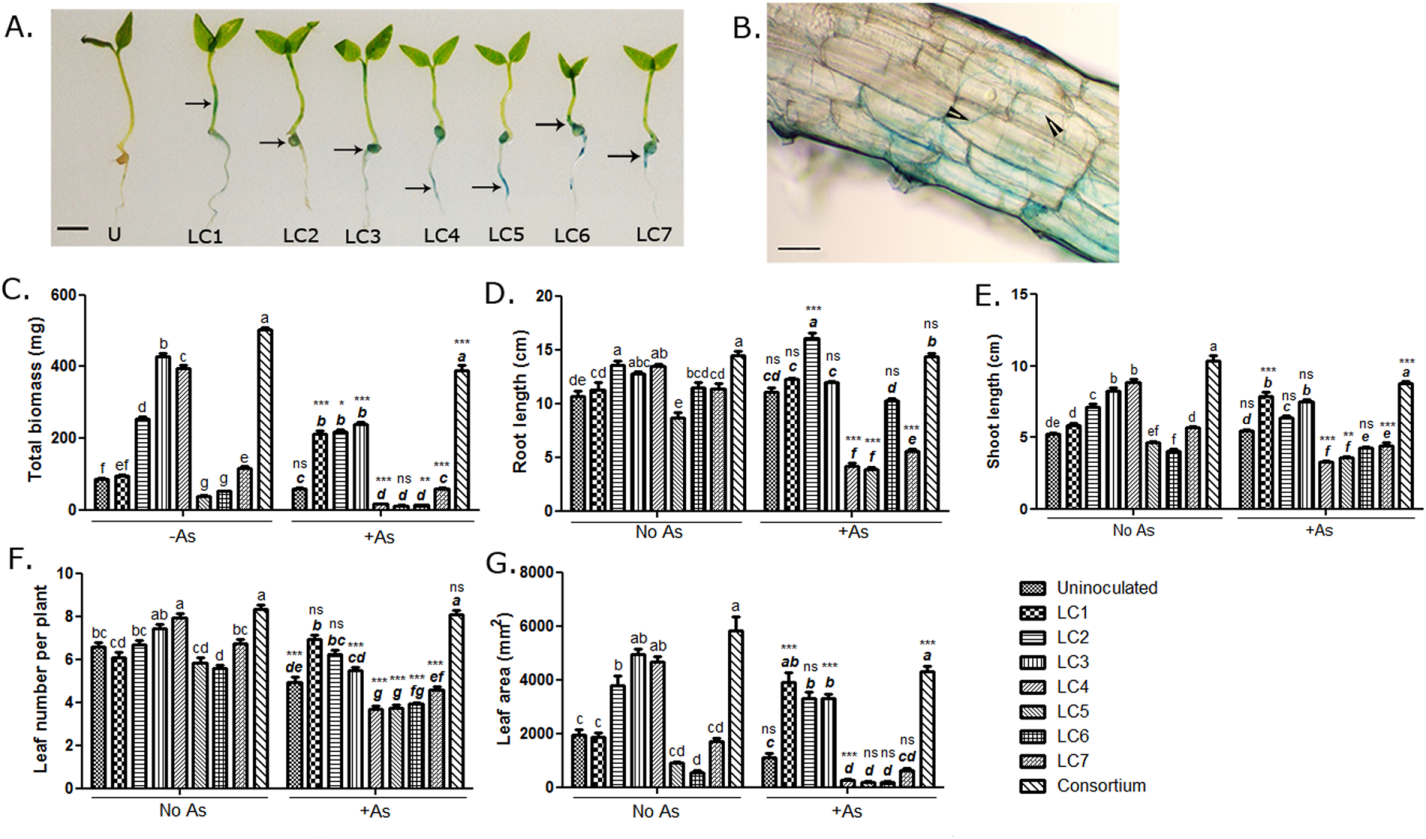
*L*. *camara* endophytes colonized *S*. *nigrum* endosphere and promoted plant growth. (**A**,**B**) One week old *S*. *nigrum* seedlings were infected with LacZ-labeled endophytes and stained with 100 µg/mL X-gal. Stained regions of plants are shown with arrows indicating colonization. LC1.:*Enterobacter* sp. LC1, LC2.:*Kocuria* sp. LC2, LC3.:*Kocuria* sp. LC3, LC4.:*Enterobacter* sp. LC4, LC5.:*Kocuria* sp. LC5, LC6.:*Enterobacter* sp. LC6, LC7.:*Kosakonia* sp. LC7. U: uninoculated control plant, Scale bar = 0.5 cm. Infected roots were visualized under a light microscope. *Kocuria* sp. LC2 colonization in root sections has been shown. Scale bar = 300 µm. Bacterial chains have been shown in arrowheads (**B**). (**C-G**) One week old *S*. *nigrum* plants were treated with 25 ppm Na_3_AsO_4_ or 1 × MS in presence or absence of the endophytes added individually or as a consortium. The biomass (**C**), root length (**D**), shoot length (**E**), leaf number (**F**) and leaf area (**G**) were determined 4wpi; n = 12. Data are represented as mean ± SEM. Bars with different letters indicate significant differences amongst different endophytic isolates at a particular As-level (bold italics for +As) obtained from two-way ANOVA with Tukey’s post-hoc test. Significant differences for an individual isolate between −As and +As treatments have been marked by *P < 0.05, **P < 0.01 ***(P < 0.001); ns = no significance. (Df for As:1, for endophytes:8, for interaction:8, error:198).

Unlike other plant-microbe interactions *viz*, plant-pathogen and legume-rhizobia interactions, endophytes show a broader host range due to relatively less influence of the host genotype on their colonization. For example, *Azoarcus* BH72, isolated from Kallar grass^36^ and nitrogen-fixing endophytes from *Typha angustifolia*^16^ were also found to colonize rice. *Rhodotorula graminis*, a basidiomycotan endophytic yeast isolated from poplar, similarly showed a broad host range^37^. Likewise, LacZ-labeled *L*. *camara* endophytes (Fig. S3A) were tested for their ability to colonize the endosphere of *S*. *nigrum*. Representative figures following X-gal staining showed blue staining in the infected plant (Fig. 2A) and bacterial colonization in the apoplastic spaces in the root (Fig. 2B). Our results confirmed that the *L*. *camara* endophytes established themselves within *S*. *nigrum* endosphere as a surrogate host (Fig. 2A,B). The endophytes also translocated systemically in the stem, node, and leaf (Figs 2A, S3B) possibly through the xylem vessels as also reported for other beneficial endophytes^38,39^. Next, the effect of the *L*. *camara* endophytes on the growth of *S*. *nigrum* seedlings was studied in the presence and absence of As. Significant interaction effects were observed between As and endophyte treatments on all the growth parameters measured by total plant biomass, root length, shoot length, leaf number and area (Two-way ANOVA; Table 2). Individually, the endophytes had differential effects on plant growth. Some had growth promoting effects both under +As and −As conditions (LC2, LC3) while some showed growth retardation (LC5, LC6; Figs 2C–G, S4). LC4 showed growth promotion in absence of As but compromised growth under +As (Two-way ANOVA with Tukey’s post-hoc test). The bacteria were also used as a consortium which ensured that the interspecies interactions that occurred in their natural environment were maintained while interacting with the plant. Multi-strain consortiums are often reported to be more effective in their PGP activity than a single bacterium^40^. Inoculation with the consortium resulted in an increase in plant height and biomass 4-weeks post infection (wpi). The growth promotion was visible even in the absence of As. Nonetheless, the difference in growth between uninoculated vs. inoculated plants was more pronounced when the plants were treated with As (Fig. 2C–G; Table 2) indicating that the consortium was most potent in increasing the vigor of *S*. *nigrum*.

**Table 2:** F-values for different plant properties due to As or endophyte (main effects) or their interaction.

Plant Properties		As-effect		Endophyte-effect		Interaction effect	
Phenotypic effects	Biomass	F(1,198) = 797.2; P < 0.0001		F(8,198) = 1040; P < 0.0001		F(8,198) = 239.7; P < 0.0001	
	Root length	F(1,198) = 134.9; P < 0.0001		F(8,198) = 115; < 0.0001		F(8,198) = 53.19; P < 0.0001	
	Shoot length	F(1,198) = 118.8; P < 0.0001		F(8,198) = 194.7; P < 0.0001		F(8,198) = 64.18; P < 0.0001	
	Leaf number	F(1,198) = 281.3; P < 0.0001		F(8,198) = 60.17; P < 0.0001		F(8,198) = 28.74; P < 0.0001	
	Leaf area	F(1,198) = 93.25; P < 0.0001		F(8,198) = 112.3; P < 0.0001		F(8,198) = 29.43; P < 0.0001	
Total chlorophyll content		F(1,32) = 300.9; P < 0.0001		F(1,32) = 258.6; P < 0.0001		F(1,32) = 24.30; P < 0.0001	
Enzyme activities		**As-effect**		**Endophyte-effect**		**Interaction effect**	
		**Root**	**Shoot**	**Root**	**Shoot**	**Root**	**Shoot**
	GR activity	F(1,36) = 39.35; P < 0.0001	F(1,36) = 157.9; P < 0.0001	F(1,36) = 6.627; P = 0.0143	F(1,36) = 1.894; P = 0.1772 (ns)	F(1,36) = 0.01235; P = 0.9121 (ns)	F(1,36) = 43.95; P < 0.0001
	GST activity	F(1,36) = 2.212; P = 0.1457 (ns)	F(1,36) = 1.198; P = 0.281 (ns)	F(1,36) = 2.435; P = 0.1274 (ns)	F(1,36) = 21.46; P < 0.0001	F(1,36) = 0.06844; P = 0.7951 (ns)	F(1,36) = 51.66; P < 0.0001
	Glutaredoxin activity	F(1,36) = 38.97; P < 0.0001	F(1,36) = 21.64; P < 0.0001	F(1,36) = 23.24; P < 0.0001	F(1,36) = 16.91; P = 0.0002	F(1,36) = 79.74; P < 0.0001	F(1,36) = 45.77; P < 0.0001
	AsPOX activity	F(1,36) = 67.44; P < 0.0001	F(1,36) = 0.1590; P = 0.6924 (ns)	F(1,36) = 27.85; P < 0.0001	F(1,36) = 0.2627; P = 0.6114 (ns)	F(1,36) = 152.0; P < 0.0001	F(1,36) = 1.329; P = 0.2566 (ns)
	POX activity	F(1,36) = 4.360; P = 0.0439	F(1,36) = 0.6863; P = 0.4129 (ns)	F(1,36) = 3.986; P = 0.0535 (ns)	F(1,36) = 9.178; P = 0.0045	F(1,36) = 7.454; P = 0.0097	F(1,36) = 20.62; P < 0.0001
	Arsenate reductase activity	F(1,36) = 106.4; P < 0.0001	F(1,36) = 97.34; P < 0.0001	F(1,36) = 2.22; P = 0.1449 (ns)	F(1,36) = 34.02; P < 0.0001	F(1,36) = 26.57; P < 0.0001	F(1,36) = 31.43; P < 0.0001

### The endophytic consortium increased arsenic phytoremediation potential of S. nigrum

**Figure 3:**
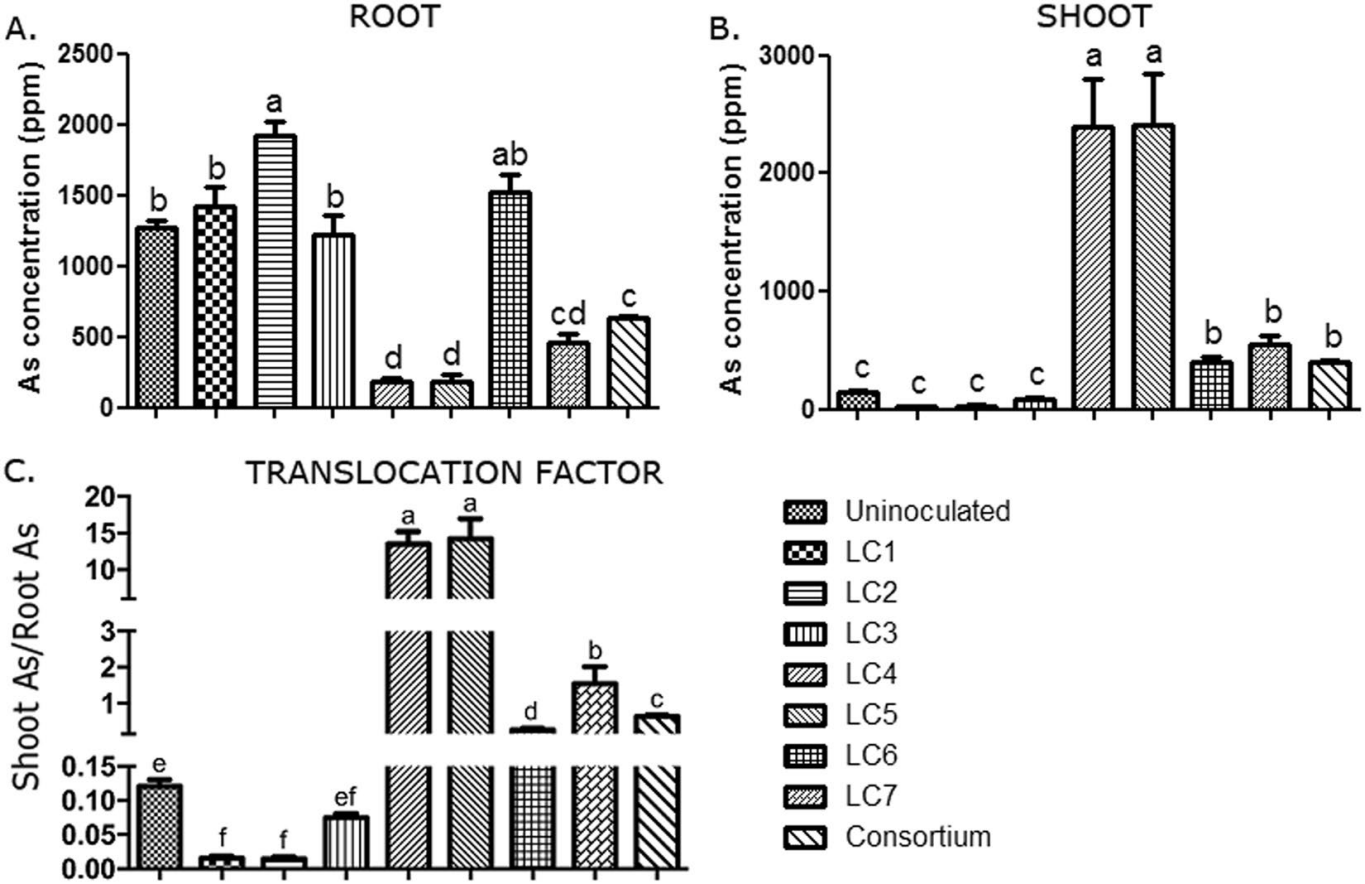
*L*. *camara* endophytes increased As accumulation and translocation in *S*. *nigrum*. *S*. *nigrum* plants were grown in presence or absence of 25 ppm As, treated with or without the endophytes. (**A**–**C**) As concentration was measured in root and shoot by ICP-OES. As concentration in plant root (**A**) and shoot (**B**), and translocation factor (shoot As/root As) (**C**) have been plotted. n = 6. Data are represented as mean ± SEM. Bars with different letters indicate significant differences amongst different endophytic isolates obtained from one-way ANOVA with Tukey’s post-hoc test.

We measured the As-concentration in plants treated with the individual microbe and the consortium under +As condition by ICP-OES. Without the endophytes, *S*. *nigrum* retained most of the As (1267 ± 56.88 ppm) in the root. LC2 increased the root retention of As. LC4, LC5, LC7 and the consortium decreased As-concentration in root (One-way ANOVA with Tukey’s HSD test) concomitantly increasing shoot As suggesting that these microbes regulated the root-to-shoot transport of As that is extremely vital for As-phytoremediation. LC4 and LC5 increased the shoot As-concentration to >2000 ppm and negatively affected plant growth which inversely correlated with shoot As-concentration. The consortium-treated plants were the only exception where both growth and root-to-shoot translocation of As increased in +As plants (Fig. 3; Table 2). The consortium also reduced As in the rhizosphere soil (Fig. S5). The ratio of shoot-As to soil-As was approximately 16 after endophyte treatment compared to *Isatis cappadocica* and *Hesperis persica* where this ratio was reported to be around 1. A 6.74-fold increase in plant biomass (Fig. 2C) suggested a significant increase in As-cleanup from the soil by endophyte-treated plants. Since the consortium appeared to be most effective in improving the potency of As-phytoremediation of *S*. *nigrum*, the endophytes were used as a consortium in the subsequent studies.

### The endophytic consortium increased photosynthetic efficiency in *S*. *nigrum* in the presence of arsenic

**Figure 4:**
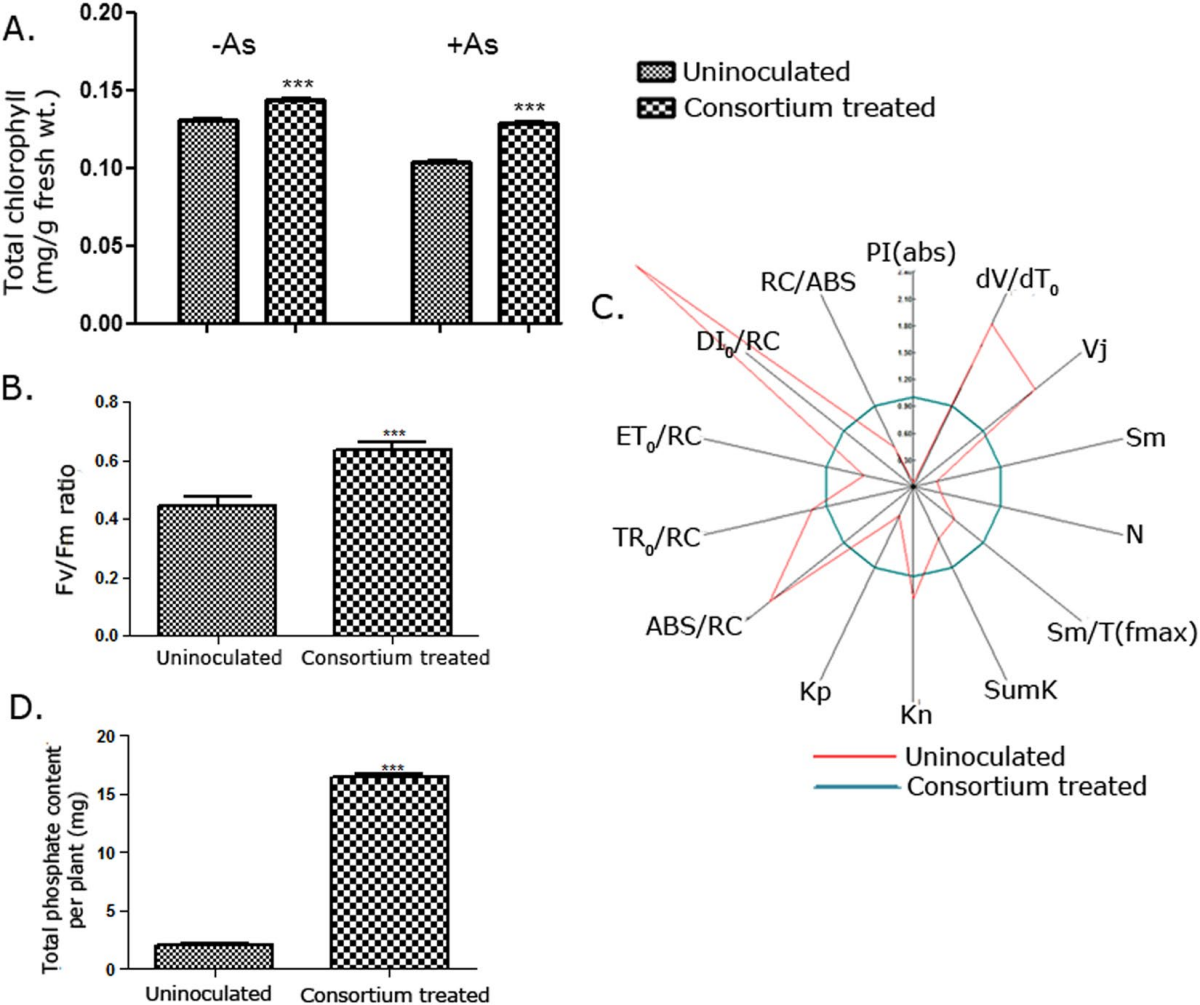
Endophyte consortium treatment improved photosynthetic efficiency and phosphate nutrition in *S*. *nigrum*. (**A**) Total chlorophyll content in plant leaves was compared between endophyte consortium-treated plants and uninoculated plants grown in the presence or absence of 25 ppm As. ***P < 0.0001 (endophyte-treated vs uninoculated; two-way ANOVA with Tukey’s post-hoc test); n = 9 (3 leaves/plant). (Df for As:1, for endophytes:1, for interaction:1, error:32). (**B** and **C**) Photosynthetic efficiency of endophyte consortium-treated plants was compared with untreated plants grown in the presence of As, represented by Fv/Fm ratio (**B**) and the radar plot (**C**). ***P < 0.0001 (unpaired two-tailed t-test); n = 9 (3 leaves/plant). (**D**) The total phosphate content of endophyte consortium treated and uninoculated plants grown in presence of 25 ppm As was measured by ICP-OES 4wpi. n = 9 (each in 3 experimental replicates). ***P < 0.0001(unpaired two-tailed t-test). Data are represented as mean ± SEM.

This increased fitness of the consortium-treated plants was further confirmed by measuring their photosynthetic efficiency. As-stress is known to decrease the photosynthetic rate in plants^41–44^. As-treated *S*. *nigrum* plants also showed leaf chlorosis and a 26.02(±0.0057)% decrease in total chlorophyll content (Figs 4A, S6). This reduction was largely restored when plants were supplemented with the consortium. The consortium increased the chlorophyll content even without As, the increase was, however, significantly greater in the presence of As [24.55(±0.0057)%] (Two-way ANOVA with Tukey’s post-hoc analysis, Table 2; Fig. 4A). The quantum efficiency (Fv/Fm) of photosystem II (PSII) increased by 1.42-times on consortium treatment (unpaired t-test; Fig. 4B). Several derived parameters were measured and are shown by a radar plot (Fig. 4C, Table S3). The results suggested that the number of photosynthetically active reaction centers was enriched by 25% in endophyte-treated plants compared to untreated control. The consortium-treated plants showed a greater PSII performance with enhanced energy flux towards electron transport, reduced energy loss by dissipation, reduction in the number of inactive reaction centers and an increase in Q_A_ turnover rate. (Fig. 4C, Table S3). This is in contrast to the reported effect of As on non-hyperaccumulator *Ceratophyllum demersum* L, where higher concentrations of As disrupted PSII performance completely^45^. Since photosynthetic performance is a direct reflection of plant growth and yield, the consortium seemed to circumvent the growth inhibition caused by As-stress imparting a fitness advantage to the plants leading to better survival in the presence of the metalloid.

### The endophytic consortium influenced several mechanisms, directly and indirectly, influencing As tolerance and/or detoxification

#### The endophytic consortium improved phosphate nutrition in S. nigrum in the presence of arsenic

Arsenate competes with phosphate for P-transporters to gain entry into the plant and thus interferes with P-sensing and responses^46^. We investigated the effect of the consortium on the total P-level of the plants. Recently, it was shown that colonization of endophytic fungus *Colletotrichum tofieldiae* in *Arabidopsis thaliana* was controlled by P-deficiency response of the plant. Phosphate was supplemented by the fungus^5^ which helped it bypass plant innate immunity. The observation that all the *L*. *camara* endophytes solubilized phosphate possibly indicated a similar nutritional requirement of the host plant under As-stress. Endophytes isolated from *Typha angustifolia* growing in the marginal wetland of a Uranium mine appeared to be primarily N-fixing^16^ and improved N-nutrition in the plant again highlighted that the endophyte properties are driven by plant nutritional status (controlled by the soil). In the presence of As, treatment with the consortium increased the total P-content per plant by about 8.05-times (unpaired t-test; Fig. 4D). Downregulation of P-transporters is a common strategy adopted by tolerant plants to bypass As-uptake^47^. However, this is likely to obstruct P-acquisition by plants affecting plant growth. The consortium improved P-nutrition of *S*. *nigrum* under As-stress and restored plant fitness in the presence of As without such a compromise. Elevated P has been reported to completely change the uptake and intracellular dynamics of As in plants^45^. Nonetheless, this improvement of P-nutrition could further increase the As-load in *S*. *nigrum*. Tu^48^ reported that supplementation of P in the growth medium indeed increased As-phytoremediation by *P*. *vittata*. The endophytes, through their effect on P-nutrition, had a two-fold effect. On the one hand, it improved plant health in presence of As, on the other hand, it increased As-phytoremediation potential.

#### The endophytic consortium enhanced reactive oxygen species generation in S. nigrum

**Figure 5:**
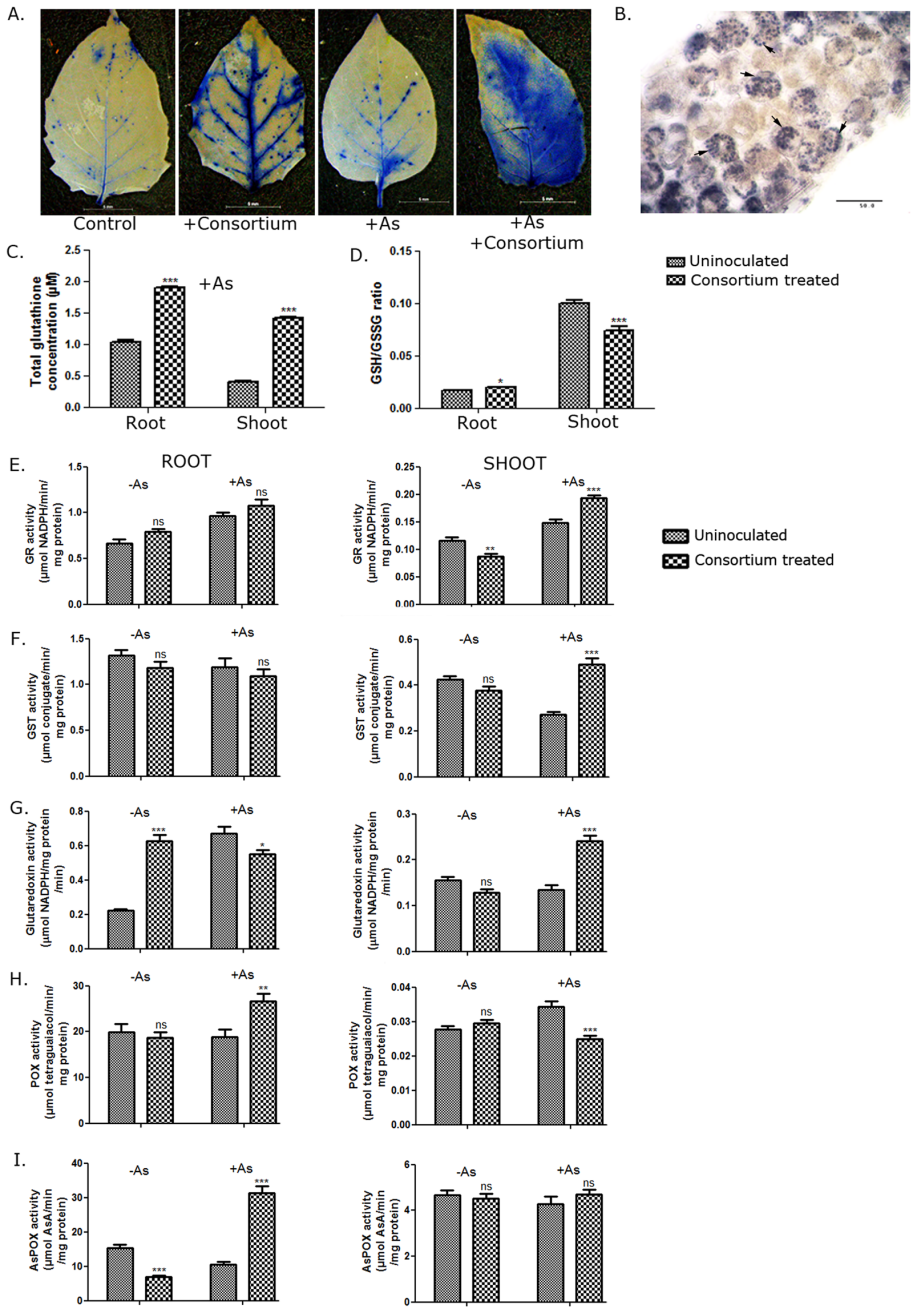
*L*. *camara* endophyte consortium augmented ROS production and anti-oxidative defense in *S*. *nigrum*. (**A**) Young leaves of endophyte consortium-treated and uninoculated plants grown in presence or absence of 25 ppm As were stained with 0.05% NBT 4wpi and visualized under a stereo microscope M205FA. Scale bar = 5 mm. (**B**) ROS production in chloroplasts of plants treated with As and endophytes. Scale bar = 50 µm. (**C** and **D**) Total glutathione content (**C**) and GSH/GSSG ratio (**D**) of endophyte consortium-treated plants were compared with untreated plants grown in presence of As. n = 14. *P < 0.05, ***P < 0.0001 (unpaired two-tailed t-test). (**E**–**I**) Antioxidant enzyme activities were measured in root and shoot of endophyte consortium-treated and uninoculated plants grown in presence or absence of As. Glutathione Reductase (GR) (**E**), glutathione S-transferase (GST) (**F**), glutaredoxin (**G**), peroxidase (POX) (**H**) and ascorbate peroxidase (APX) activities (**I**) were plotted using GraphPad Prism5. n = 10 (each in 2 experimental replicates). *P < 0.05, **P < 0.01, ***P < 0.0001, ns = no significance (endophyte-treated vs uninoculated; Two-way ANOVA with Tukey’s post-hoc test). Data are represented as mean ± SEM. (Df for As:1, for endophytes:1, for interaction:1, error:36).

A major cause of As-induced cell damage is believed to be oxidative stress^49,50^. Several detoxification strategies in plants are known to contribute towards As-tolerance. These processes were investigated in consortium-treated plants to understand the mechanism of consortium-mediated As-detoxification. Leaves of 4-week old *S*. *nigrum* plants grown in the presence and absence of As, with and without the consortium, were stained with Nitroblue tetrazolium (NBT) that stains superoxide radicals^51^. The consortium-treated plants showed intense staining even in absence of As (Fig. 5A). Staining was noted around the veins in plants treated with only As or the consortium (Figs 5A, S7A left panel). ROS generated due to the accumulation of uncomplexed As(III) is reported to accumulate in plant veins^52^. The pattern of superoxide accumulation noticeably changed upon As + consortium treatment. The staining became most intense and occurred throughout the leaf (Figs 5A, S7A right panel). Whether this change in the ROS-staining was due to change in As-distribution is difficult to predict, however, microscopic studies showed that the staining occurred in the chloroplasts (Fig. 5B). Plant ROS responses occur in distinct organelles like chloroplast, mitochondria, peroxisome, apoplast and the nucleus. Depending on the site of production, ROS has specific signatures and distinct biological readouts. For example, ROS production in chloroplast is implicated in signaling response and appears to be distinct from peroxisome where the ROS production triggers repair^53^. The ROS production was also highest in roots of As + consortium-treated plants, where the staining pattern appeared to be distinctly different from As-treated plants; probably indicating that different organelles participate in ROS production (Fig. S7B). The endophytes, therefore, appeared to contribute to sustained ROS generation in plants without having any deleterious effect on the plant health. This was clearly different from the ROS generation in plants that occur as an immediate response to its encounter with microbe-associated-molecular-patterns (pathogenic or symbiotic)^54^. Recently it has been suggested that ROS is not necessarily indiscriminately cytotoxic as believed earlier, but rather functions in cellular surveillance and signaling^55,56^. A balance between ROS production and induction of anti-oxidative defense is central to acclimation to abiotic stress. Beneficial interaction of plants with arbuscular mycorrhiza and *Piriformospora indica* is known to confer cross-tolerance to a variety of abiotic and biotic stressesand requires the generation of ROS^57^. *P*. *indica* reduced As induced root damage and As translocation in rice^58^. Total glutathione level was increased 1.8-times in root and 3.4-fold in the shoot (Fig. 5C) in the presence of As + consortium compared to As-treated plants (unpaired t-test). Although the total glutathione content increased, the reduced (GSH) to oxidized glutathione (GSSG) ratio was decreased (Fig. 5D) further confirming more ROS generation in As + endophyte plants.

#### The endophytic consortium elicits distinctly different oxidative defense response in root and aerial tissues

Enhanced glutathione biosynthesis and metabolism is an accepted strategy that improves As tolerance in plants^59^. Glutathione influences the activity of many enzymes involved in detoxification, sequestration, and transport of As directly or indirectly^60^. Activities of enzymes that regulate the reduced glutathione pool or indirectly depend on the glutathione pool were tested. These were glutathione reductase (GR) that catalyzes the formation of GSH from GSSG, glutaredoxin and glutathione S-transferase (GST). Glutaredoxins receive electrons from glutathione and donate them to oxidized substrates. Overexpression of glutaredoxins has been reported to increase As-tolerance in plants^61,62^. GSTs transfer glutathione moieties to xenobiotics for detoxification. Activities of all three ‘glutathione-related’ enzymes were increased in shoots of As-treated plants specifically upon consortium treatment (Fig. 5E–G) with significant interaction effects between As and endophytes (Two-way ANOVA, Table 2). The consortium appeared to expand the glutathione pool and influenced glutathione-related detoxification mechanisms without the requirement of any transgenic approach long sorted to improve As-resistance in plants. Activities of these enzymes were however not significantly altered in roots. In contrast, activities of ascorbate peroxidase (APX) and peroxidase (POX) were enhanced in As-treated plant roots (Fig. 5H,I) rather than shoots upon endophyte treatment with significant interaction effects between As and endophytes (Two-way ANOVA, Table 2) indicating that As-mediated upregulation of anti-oxidative defense was affected by the consortium. Peroxidases are components of the ascorbate-glutathione cycle that constitute the most prominent ROS detoxification system in plants. Peroxidase activity scavenges H_2_O_2_ and is almost always associated with a concomitant increase in GR activity. Interestingly, it was not so in our case. Peroxidase and GR activities were antagonistic in root and shoot indicating additional layers of tissue-specific regulation. In the shoot, increased As-content as a consequence of endophyte treatment probably triggered glutathione-mediated detoxification. In contrast, more peroxidase activity implied more H_2_O_2_ generation in roots which were scavenged or potentially participated in signaling responses. In summary, the endophytes boosted both the non-enzymatic and enzymatic antioxidant defense in an As-dependent manner in the plant and this together with heightened ROS-levels provided enhanced protection against As-stress.

#### The endophytic consortium increases As(V) to As(III) conversion in plants

**Figure 6:**
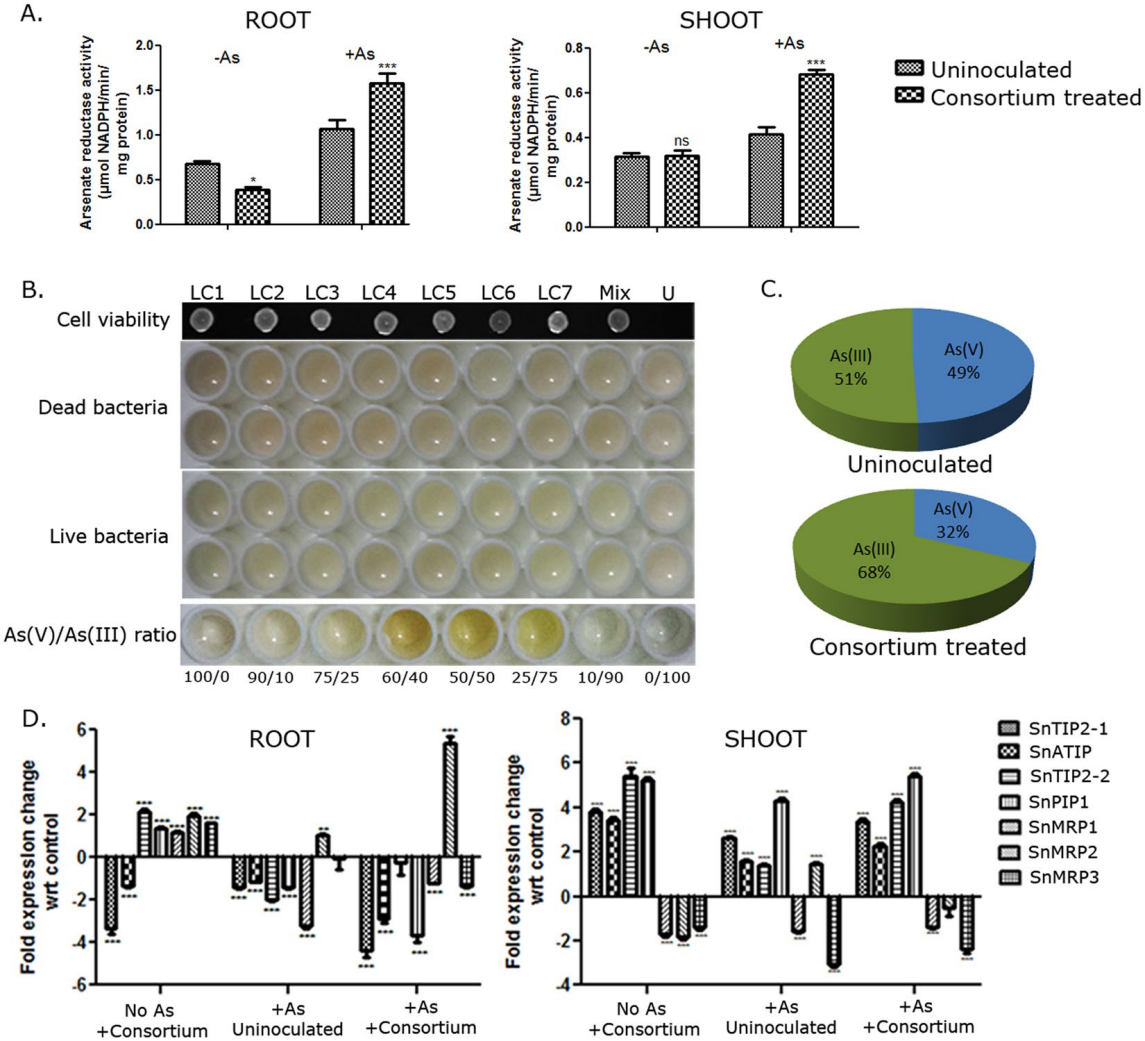
*L*. *camara* endophyte consortium reduced arsenate, upregulated arsenate reductase activity in As- dependent manner and differentially regulated aquaporin and MRP genes. *S*. *nigrum* plants were grown in presence or absence of 25 ppm As, treated with or without the endophytes used as a consortium. (**A**) Arsenate reductase activity was measured in the root and shoot 4wpi. n = 10 (each in 2 experimental replicates). *P < 0.05, ***P < 0.001 (endophyte- treated vs uninoculated; Two-way ANOVA with Tukey’s post-hoc test). (Df for As:1, for endophytes:1, for interaction:1, error:36). (**B**) AgNO_3_ was added to bacterial cells (live & dead) incubated in Tris-Cl buffer with arsenate for 48 h and the colour was compared to standards having varying As(V)/As(III) ratio. Viability of live cells was measured. LC1.:*Enterobacter* sp. LC1, LC2.:*Kocuria* sp. LC2, LC3.:*Kocuria* sp. LC3, LC4.:*Enterobacter* sp. LC4, LC5.:*Kocuria* sp. LC5, LC6.:*Enterobacter* sp. LC6, LC7.:*Kosakonia* sp. LC7., Mix: Consortium, U: uninoculated control. (**C**) As(V) and As(III) speciation was performed in *S*. *nigrum* shoot extracts grown in the presence or absence of the consortium. As(V) and As(III) was separated using calcium alginate beads followed by ICP-OES. Results have been represented as pie charts showing the percentage of each species in the total As. n = 6. (**D**) cDNA was prepared from root and shoot of 4-week infected plants and expression of 4 aquaporins and 3 MRP transporters in root and shoot were measured by real-time PCR. The fold change of expression has been normalized to that of control. SnTIP2-1, SnATIP and SnTIP2-2 = Tonoplast intrinsic proteins; SnPIP1 = Plasma membrane intrinsic protein; SnMRP1, SnMRP2 and SnMRP3 = Multidrug resistance-associated proteins. *P < 0.05,**P < 0.01,***P < 0.001. Data are represented as mean ± SEM.

As(V) to As(III) conversion is an important mechanism for As-detoxification. As(III) forms stable complexes with thiol-containing compounds, glutathione, and glutathione-derived peptides− phytochelatins (PCs)^63^ and are immobilized in vacuoles by MRP transporters. Arsenic is transported to the shoot via aquaporin proteins as As(III), which is the preferred form of As for phytoremediation^64^. As(III) can also be extruded out of plant cell^64–67^. The activity of arsenate reductase catalyzing As(V) to As(III) conversion was increased both in shoot and root of consortium-treated plants in an As-dependent manner (Two-way ANOVA, Table 2; Fig. 6A). Overexpression of bacterial arsenate reductase and gamma-glutamylcysteine synthetase conferred greater As-tolerance^59^. The endophytic consortium expanded the glutathione pool and simultaneously increased arsenate reductase activity, mimicking an analogous physiological state in *S*. *nigrum*. The endophytes were individually tested for their ability to convert As(V) to As(III) using the ability of AgNO_3_ to provide specific color (yellow or orange) to As(V) and As(III) mixed in a given ratio^68^. All the endophytes (but not the dead bacteria) individually or as a consortium changed the color of AgNO_3_ in a way suggesting a 25–40% conversion of As(V) to As(III) (Fig. 6B). Arsenic speciation in *S*. *nigrum* shoot extracts was performed by separating As(V) from As(III) utilizing the selective ability of As(V) to bind Fe-doped Ca-alginate beads at pH3 followed by ICP-OES^69^. pH had negligible effect on As(V) to As(III) conversion (data not shown). Our results revealed that *S*. *nigrum* had an innate ability to convert As(V) to As(III). 51% of As(V) was converted to As(III) in untreated plants. The As(III) increased to 68% when the plants were treated with the consortium (Fig. 6C). This confirmed that the endophytes reduced As and potentially contributed to the long-distance transport and detoxification of As.

This results led us to investigate the expression of aquaporin-like proteins which play important role in the transport of As(III)^70,71^ and H_2_O_2_ across membrane^72^. Expression of three *S*. *nigrum* tonoplast intrinsic proteins (TIPs) and one plasma membrane intrinsic protein (PIP) was checked by real-time PCR. In roots, SnTIP2-2 (GU594261) and SnPIP1 (GU575314) were upregulated by 2.1 and 1.4-fold respectively, and SnTIP2-1 (GU594266) and SnATIP (GU594268) were downregulated by 3.3 and 1.3-fold respectively upon endophyte treatment. In the presence of As + consortium, the downregulation of SnTIP2-1 and SnATIP was further pronounced (4.4 and 3.9-fold respectively) (Fig. 6D). Although upregulated on consortium treatment, SnPIP1 was strongly downregulated (3.7-fold) when As was together with the consortium. The endophytes upregulated all four aquaporins in the shoot. Arsenic treatment alone also led to upregulation of the aquaporins in shoots, but at a much lower level compared to the consortium -treated plants.

Further, expression of MRP transporters was studied in As and/endophyte treated plants. MRP transporters are known for sequestration of As-PC complexes in plant vacuoles and are reported to be upregulated in response to As-stress^73^ which make them important candidates for studying As detoxification. Unlike the aquaporins, no sequences for *S*. *nigrum* MRPs were available in the nucleotide database. Therefore three partial MRP sequences SnMRP1 (1015 bp, KY448289), SnMRP2 (959 bp, KY448290) and SnMRP3 (634 bp, KY448291) (Fig. S8A–C) homologous to *Arabidopsis thaliana* AtABCC1 and AtABCC2^73^ were cloned from *S*. *nigrum*. The putative SnMRPs belonged to three phylogenetically distinct classes. Among these, in the root, SnMRP1 was upregulated upon consortium treatment but downregulated by 3-fold upon As-treatment. SnMRP2 showed the most interesting regulation. It was strongly upregulated (5-fold) in root upon endophyte treatment exclusively in presence of As. In the shoot, SnMRP2 was upregulated (1.5-fold) only in presence of As. Endophyte consortium treatment downregulated the expression of all the other MRPs in the shoot (Fig. 6D). Differential regulation of these genes may indicate potentially important roles played by them in controlling plant’s response towards As and/or the consortium.

## Conclusion

The success of microbe-assisted phytoremediation is dictated both by the plant as well as the microbes under question. Both the partners, therefore, need to be carefully chosen for an efficient outcome. Our work shows that an endophytic consortium isolated from a plant collected from an As-contaminated site could be used in conjunction with an accumulator plant to improve its As-phytoremediation ability. The consortium but not the individual microbes effectively enhanced both plant growth and root to shoot transport of the metalloid- a tightly controlled property of a plant. As a plant model, *S*. *nigrum* was found suitable for removing low concentration of As present in the soil of many highly populated areas. The consortium improved P-nutrition and enhanced ROS/oxidative defense in the plant indirectly affecting plant fitness under stress. Simultaneous *in planta* increase in As(V)-As(III) conversion was also observed that could have a direct influence on As long-distance transport and detoxification. SnMRP2, an MRP gene from *S*. *nigrum*, was specifically upregulated upon endophyte treatment in an As-dependent manner. Metal hyperaccumulator plants are often slow growing. Their use in fields is also constrained by their adaptation to particular geographical areas. This work demonstrates that a suitable plant model used as surrogate host together with endophytes with the appropriate properties can widen the use of endophyte-assisted phytoremediation in the clean-up of soil without such restrictions.

## Methods

### Isolation and identification of endophytes from *Lantana camara*

*Lantana camara* shoot samples were collected from Nonaghata-Dasdia block in Nadia district of West Bengal (Latitude = 23°14′45.5′′N, Longitude = 88°36′49.5′′E), India. Plant tissues were washed with tap water and then in a sonicating water-bath with sterile water to remove adhered soil particles and epiphytic microbes. Samples were surface sterilized as described in Saha *et al*.^16^. The last wash was plated on Tryptone Soy Broth (TSB, Himedia) agar plates as a negative control. Surface sterilized shoot tissue samples were ground and were extracted in 0.1(M) phosphate buffer (pH 7.0). Fifty microliters of the extract were plated on TSB plates supplemented with 500 ppm Na_3_AsO_4_ (As henceforth). The plates were incubated at 30 °C for three days. Colonies having distinct morphologies were subcultured on TSB plates containing 500 ppm As to obtain pure cultures. 16S rDNA was amplified from the genomic DNA of the endophytes using universal primers 27F & 1492R^74^, cloned into pJET1.2 Blunt vector (Thermo-Scientific) and sequenced. Bacterial nomenclature was done based on their closest homologs in the EZBioCloud database of the prokaryotic type strain. The 16S rDNA sequences were subjected to BLAST analysis against NCBI database.

### Bacterial IAA production

IAA was estimated by Salkowsky’s Method^75,76^. Endophyte cultures were grown in Luria-Tryptophan broth with (1000 ppm) or without As and incubated at 30 °C for 48 h. Four milliliters of Salkowsky reagent was added to 1 mL of the culture supernatants. After 30 min of incubation at room temperature, IAA production was quantified against a standard curve by measuring the absorbance at 540 nm. Experiments were done twice with 9 replicates each (n = 18).

### Estimation of inorganic phosphate solubilization

Bacterial cultures were inoculated in Pikovskaya’s (PVK) broth^77^ with (500, 1000 ppm As) or without As at 30 °C for 5 days. The amount of phosphate (P) solubilized in the culture supernatant was estimated by comparison with a standard curve for soluble P (0.5 ppm–30 ppm) prepared by reacting K_2_HPO_4_ with acid molybdate-ascorbic acid reagent. The absorbance of the complex was measured at 880 nm^78^ and represented as solubilized phosphate per gram of bacterial cells. The P-concentration in the uninoculated sample supernatant was subtracted from the test. Experiments were done twice with 6 replicates of each treatment. For qualitative P-estimation, 4 µL of culture from each isolate at O.D_600_ = 0.4 was spotted on PVK agar in presence or absence of 1000 ppm As. The plates were incubated at 30 °C for 5 days and scored for halo production. The experiment was done twice in triplicate.

### Labeling of endophytes with LacZ construct

Antibiotic resistance of each endophyte was determined using Kirby-Bauer Disk Diffusion Susceptibility Test. *E*. *coli* SM10λpir was transformed with pRJPaph-lacZYA plasmid. The plasmid was mobilized from the donor to the recipient endophytes by filter-mating technique^79^ and the transconjugants were selected on TSB media containing the plasmid (10 µg/mL tetracycline) and endophyte-specific (Table 1) antibiotics. LacZ-labeling was confirmed by X-gal overlay assay. Plants infected with labeled endophytes were stained with X-gal according to the protocol described by Ledermann *et al*.^80^. Stained plant root sections were observed under a bright-field microscope (Dewinter, Biowizard). Pictures of leaves were taken using a stereo-microscope (Leica M60).

### Plant growth conditions, treatment, and measurement of growth promotion

*Solanum nigrum* seedlings were germinated on filter paper. One week old seedlings of equal lengths were transferred into pots (8 cm diameter, 15 cm height) containing 32 g of artificial soil (SoilRite^TM^) mixed with 10 mL of 1X Murashige and Skoog’s (MS) salt solution (pH 5.8) with or without 25 ppm As. Arsenic was applied only once. The plants were grown in a climate controlled room at 25 °C under 16/8 h light/dark conditions. Whenever required, 1X MS salt solution (pH 5.8) was supplied in equal amounts to all plants. The endophytic consortium was prepared by suspending 10^7^ cfu of each microbe in 10 mL 1X MS and was applied to the soil only once around plant root post-transplantation. Different experiments were set up with different sets of plants grown to conduct analyses like plant growth parameters, chlorophyll content, photosynthetic efficiency, As-content, P-content, glutathione, and enzyme assays. Plants were harvested 4wpi. Root-length, shoot-length, leaf number, leaf blade area, and total dry biomass were measured as plant growth parameters (two independent experiments with 6 replicates each, was performed n = 12). The effect of the consortium on plant growth vs. uninoculated control was confirmed using n = 24 plants in 3 independent experiments with similar results (data not shown).

### Chlorophyll estimation in *S*. *nigrum* leaves

400 mg fresh leaf samples were extracted overnight in 7 mL 80% acetone and the absorbance at 645 nm and 660 nm was measured using SHIMADZU UV-2450 UV-visible spectrophotometer. Total chlorophyll was calculated according to Sweeney and Martin^81^. The experiment was conducted using n = 9 plants (3 leaves/plant) per treatment.

### Estimation of photosynthetic parameters in *S*. *nigrum*

Photosynthetic parameters were measured by HandyPEA chlorophyll fluorimeter (Hansatech instruments) as per manufacturer’s instructions. Results were analyzed using Biolyzer 4HP v3.06 software and represented as a radar plot^82^. The result shown is an average of parameters of 3 leaves from n = 9 plants (per treatment).

### Arsenic estimation in soil and plant samples by Vapour Generation Assembly-Atomic absorption spectrometry (VGA-AAS)

Plant tissues collected from the contaminated site were washed thrice in a sonicating water-bath with 30 s pulses. Oven dried (60 °C for 48 h) plant and soil samples were pulverized and 100 mg of plant samples were digested with 2.5 mL concentrated HNO_3_ (65% Merck, Germany) and 2.5 mL H_2_O_2_ (30%, Merck, Germany)^24^. 500 mg soil samples were digested with 6:1:2 concentrated HNO_3_ (suprapur, 65%, Merck, Germany): perchloric acid (60%, Merck, Germany): HF (40%, Merck, Germany). Digested samples were filtered with Whatman 110 nm filter paper, and the total As concentration was determined using VGA-AAS (Varian SpectrAA).

### ICP-OES for estimation of total phosphate and arsenic in plant tissue

100 mg of total plant samples were digested as described. Digested samples were filtered with Whatman 110 nm filter paper followed by a 0.22 µm filter. The total P or total As-content in the digest was measured by ICP-OES (ICAP duo 6500, Thermo-Scientific, RF power 1150 watt, pump rate 50 rpm, auxiliary gas flow 1 L/min, nebulizer gas flow 0.6 L/min and coolant gas flow 12 L/min). NIST Standard Reference Material 1645 digested in the same way as the experimental samples were used as a standard for P-estimation. The experiment was conducted with n = 9 plants each in triplicate for phosphate estimation. For total As estimation after individual bacterial treatment n = 6 plants were used for individual endophytes in 2 independent experiments. Although results for n = 6 plants are presented, the effect of the consortium vs. uninoculated control was studied in n = 18 plants in 3 independent experiments with similar results.

### NBT staining

O_2_^−^ production was detected *in situ* by vacuum-infiltrating leaf and root samples with 0.05(M) sodium phosphate buffer containing 0.05% NBT for 15 min followed by incubation for another 45 min. Leaf tissues were subsequently transferred into absolute alcohol for de-chlorophyllization and imaging was done under a Leica stereo microscope M205FA equipped with a Leica DFC310FX digital camera (Leica Microsystems). Plant sections were studied under a bright field microscope (n = 10).

### Glutathione measurement in plant tissues

Total glutathione content and the GSH/GSSG ratio in plant tissues were determined by Oxiselect^TM^ Total Glutathione estimation kit (Cell Biolabs, INC) as per the manufacturer’s instructions. The GSH level was obtained without adding the enzyme glutathione reductase into the reaction mix. The GSH level was subtracted from the total glutathione level to obtain GSSG level in the samples. Experiments were done with leaf extracts from 14 independent plants each in 3 replicates.

### Enzyme assays in plants

Plant root and shoot tissues (100 mg) were ground in liquid nitrogen and extracted in phosphate buffer (pH 7.5) containing 1 mM PEG 8000, 1 mM PMSF, 8% PVP (w/v) and 0.01% (v/v) Triton X-100. Homogenates were centrifuged and supernatants were used for protein estimation (Bradford, 1976). APX, POX, GR and GST activities were measured according to Venisse, *et al*.^51^ with the following modifications. For APX 10 µL of the extract, for GR 5 µL of the extract was added to 1 mL of the reaction mixture. For determination of GST activity, 5 µL of the plant extract was added to 100 µL and for POX 50 µL of the plant extract was added to 200 µL of the reaction mixture. Arsenate reductase assay was performed according to Cesaro *et al*.^83^. 5 µL of the plant extract was added to a 100 µL of the assay mixture. Glutaredoxin assay was performed according to Verma, *et al*.^62^. 5 µL of plant extract was added to 100 µL of an assay mixture Experiments were done with leaf extracts from 10 independent plants each in 2 experimental replicates. Change in absorbance was noted at 39 s and 117 s time points.

### Arsenate reduction activity measurement in bacteria

Arsenate reduction by the endophytes was assayed by colorimetric analysis of the reaction between As(V) or As(III) and AgNO_3_. Saturated cultures were diluted to O.D._600_ = 0.6, centrifuged and the pellets were dissolved in 1 mL of 0.2 mM Tris-Cl buffer (pH7.4). 20 µL of cultures were mixed with 80 µL of 0.2(M) Tris-Cl (pH7.4) and 0.67 mM tri-sodium arsenate and incubated for 48 h at 30 °C. 100 µL of 200 mM AgNO_3_ was added for color development and compared with standards containing known ratios of As(V) and As(III). Autoclaved bacteria were used as dead bacterial controls. The viability of live cells post-incubation was checked on TSB plates. The experiment was done in 3 biological replicates (bacteria grown three times), each in triplicate.

### Arsenic speciation in plant extracts

Frozen *S*. *nigrum* shoot was finely ground in liquid nitrogen and extracted in 10 mL water degassed with argon. The extracts were again degassed for 15 mins followed by shaking for 18 h. Samples were centrifuged and total As was measured in the supernatant by ICP-OES (ICAP duo 6500, Thermo-Scientific). The pH of the supernatant was adjusted to 3.0 and was incubated with Fe^3+^-doped calcium alginate (Fe-CA) beads for 20 mins under shaking conditions. The method of preparation of Fe-CA beads has been described elsewhere^84^. It has been decisively shown that at pH3, only As(V) was quantitatively absorbed by Fe-CA beads^69^. Therefore, after incubation with Fe-CA beads only As(III) remained in the solution, which was measured by ICP-OES and compared with the As concentration of the initial solution. As(V) concentration was calculated by subtracting the As(III) content from total As. The As species have been represented as percentage of total As content. The experiment was performed with six independant plants per treatment.

### Cloning of MRP genes from *S*. *nigrum*

tBLASTn search was performed using AtABCC1 (AF008124.1) and AtABCC2 (AF014960.1) sequences as a query against NCBI nr database choosing *Solanum* sp. as an organism. Sequences obtained were aligned and primers were designed from the conserved regions of MRPs. A primary PCR followed by nested PCR was performed using primer sequences and conditions mentioned in Table S4. Primary PCR products for SnMRP1 and SnMRP2 and nested PCR product for SnMRP3 were cloned into pJET1.2 cloning vector (Thermo-Scientific) and sequenced.

### Gene expression analysis with Real-time PCR

Total RNA was isolated from the frozen tissues of *S*. *nigrum* using Plant and Fungal RNA isolation Kit (Himedia, India) and was subjected to DNaseI digestion (0.5 U/µg RNA). 2 µg RNA was used to synthesize cDNA with RevertAid Reverse Transcriptase (Fermentas) and Random Hexamer Primer (Fermentas, Waltham, MA, USA) as per the manufacturer’s protocol. To study expression changes of aquaporins and MRP transporters, cDNAs from arsenic and/or endophyte-treated (3wpi) plants were used along with untreated controls. A 10 µL qRT-PCR reaction mix contained 5 µL DyNAmoColorFlash SYBR GreenI master mix (Thermo-Scientific), 0.5 µM of each primer with 50 ng cDNA for shoot tissue and 100 ng cDNA for root tissue. Real-time PCR was conducted (Step one plus, Applied Biosystems) using a thermal step-up as follows: 10 min at 95 °C, followed by 40 cycles of 10 s at 95 °C, 15 s at 60 °C and 30 s at 62 °C. Gene expression fold changes of treated plants relative to controls were calculated by the 2^−ΔΔCt^ method. Experiments were conducted in three biological replicates (independently isolated RNA), each in triplicates. Primer sequences are listed in Table S3. Primer efficiencies were checked using LinRegPCR version 2016^85^.

### Statistical analysis

All graphs were plotted using GraphPad Prism 5.02 (GraphPad Software, Inc., La Jolla, CA, USA). All statistical analyses were performed using SPSS13 (IBM Corp.). Endophyte PGP activities, plant phenotypes (biomass, root and shoot length, leaf number, and leaf area), chlorophyll content, and antioxidant enzyme activities, and arsenate reductase assay (plant) were statistically analyzed using two-way ANOVA. Normal distribution of our data was confirmed by Shapiro-Wilk test and histogram analyses prior to performing ANOVA. The sample sizes compared being same, the assumption of homogeneity of variance was overlooked given that ANOVA is quite robust to heterogeneity of variance in such cases. The two-way ANOVA models comprised both main as well as interaction effects, and the corresponding F-tests had appropriate degrees of freedom depending on the total number of samples. Tukey’s post-hoc comparisons were performed among different endophytes at each As-level as well as among different As-levels for each endophyte treatment. As-content in plant tissues and translocation factor were compared among the different endophyte isolates using one-way ANOVA with Tukey’s HSD comparisons. For plant phosphate measurement and glutathione estimation, since the comparison was performed across two groups viz. control and endophyte consortium only under + As treatment, unpaired two-tailed t-test was performed.

### Data availability statement

All experimental data and unedited images are available with the authors.

## Electronic supplementary material

Supplementary information
