# Author Correction: An Endophytic Bacterial Consortium modulates multiple strategies to improve Arsenic Phytoremediation Efficacy in *Solanum nigrum*

**DOI:** 10.1038/s41598-018-33707-1

**Published:** 2018-10-31

**Authors:** Gairik Mukherjee, Chinmay Saha, Nabanita Naskar, Abhishek Mukherjee, Arghya Mukherjee, Susanta Lahiri, Arun Lahiri Majumder, Anindita Seal

**Affiliations:** 1 Department of Biotechnology, Dr. B. C. Guha Centre for Genetic Engineering and Biotechnology, University of Calcutta, 35, Ballygunge Circular Road, Kolkata, 700019 India; 2 Department of Endocrinology & Metabolism, Institute Of Post Graduate Medical Education & Research and SSKM Hospital, Room No. 9A, 4th Floor, Ronald Ross Building, 244, AJC Bose Road, Kolkata, 700020 India; 3 Department of Environmental Science, University of Calcutta, 35, Ballygunge Circular Road, Kolkata, 700019 India; 4 Saha Institute of Nuclear Physics, Sector - 1, Block - AF Bidhannagar, Kolkata, 700064 India; 5 Homi Bhabha National Institute, 1/AF Bidhannagar, Kolkata, 700064 India; 6 Division of Plant Biology, Bose Institute, P-1/12 CIT Scheme VIIM, Kolkata, 700054 India

Correction to: *Scientific Reports* 10.1038/s41598-018-25306-x, published online 03 May 2018

The Acknowledgements section in this Article is incomplete.

“We thank Prof. N.D. Paria, University of Calcutta (CU) for help in plant identification. We thank Dr. Hans-Martin Fischer (ETH, Zurich) for the pRJPaph-LacZYA plasmid. We also thank Dr. Senjuti Sinha-Roy (NIPGR, India), Dr. Geetanjali Sundaram (CU), Dr. Krishna Ray (WB State University), Dr. Sourabh Ghosh (ISI, India) and Dr. Soumen Manna (SINP, India) for insightful discussions and Monica Ghosh for assisting in microbe labeling. We thank Jini Shirlaw-Seal for editing the manuscript. We acknowledge Prof. I.B. Chatterjee, Dr. Maitrayee DasGupta and DBT-IPLS, CU for infrastructural help. Lastly, we acknowledge DBT, Govt. of India, project No. BT/PR15410/BCE/08/861/2011 for funding. The ICPOES work was carried out at SINP-DAE, Govt. of India 12 five-year plan project Trace, Ultratrace Analysis, and Isotope production.”

should read:

“We thank Prof. N.D. Paria, University of Calcutta (CU) for help in plant identification. We thank Dr. Hans-Martin Fischer (ETH, Zurich) for the pRJPaph-LacZYA plasmid. We also thank Dr. Senjuti Sinha-Roy (NIPGR, India), Dr. Geetanjali Sundaram (CU), Dr. Krishna Ray (WB State University), Dr. Sourabh Ghosh (ISI, India) and Dr. Soumen Manna (SINP, India) for insightful discussions and Monica Ghosh for assisting in microbe labeling. We thank Jini Shirlaw-Seal for editing the manuscript. We acknowledge Prof. I.B. Chatterjee, Dr. Maitrayee DasGupta and DBT-IPLS, CU for infrastructural help. We acknowledge DBT, Govt. of India, project No. BT/PR15410/BCE/08/861/2011 and UGC-UPE II grant of CU for funding. The ICPOES work was funded by Govt. of India 12 five-year plan project Trace, Ultratrace Analysis, and Isotope production, SINP-DAE.”

