# Supplementary material for: An Endophytic Bacterial Consortium modulates multiple strategies to improve Arsenic Phytoremediation Efficacy in Solanum nigrum
Source: Sci Rep. 2018 May 3;8:6979. doi: 10.1038/s41598-018-25306-x (PMC5934359; doi:10.1038/s41598-018-25306-x)
Supplement: Supplementary file 1 — Supplementary information [file 41598_2018_25306_MOESM1_ESM.pdf]

Supplementary Information

An Endophytic Bacterial Consortium modulates multiple strategies to improve Arsenic Phytoremediation Efficacy in *Solanum nigrum*

Gairik Mukherjee[1], Chinmay Saha[2¥], Nabanita Naskar[3,4¥], Abhishek Mukherjee[5], Arghya Mukherjee[1], Susanta Lahiri[4], A. Lahiri Majumder[5], Anindita Seal[1*]

Supplemental Tables

TableS1. Antibiotic sensitivity of endophytic isolates for biparental mating

Isolate	Antibiotic resistance shown
Enterobacter sp. LC1	Azithromycin (5µg/mL), Ampicillin (100µg/mL)
Kocuria sp. LC2	Azithromycin (5µg/mL), Ampicillin (100µg/mL)
Kocuria sp. LC3	Azithromycin (5µg/mL)
Enterobacter sp. LC4	Carbenicillin (100µg/mL)
Kocuria sp. LC5	Carbenicillin (100µg/mL)
Enterobacter sp. LC6	Azithromycin (5µg/mL), Ampicillin (100µg/mL)
Kosakonia sp. LC7	Carbenicillin (100µg/mL)

TableS2.Fold changes of different growth parameters and As-translocation factor upon Endophyte treatment (vs no Endophyte control).

	**Biomass**		**Root length**		**Shoot length**		**Leaf number**		**Leaf area**		**% survivors relative to control (+As; n=21)**	**As translocation factor**
	**-As**	**+As**	**-As**	**+As**	**-As**	**+As**	**-As**	**+As**	**-As**	**+As**		
**LC1**	no change	Increase, 3.7-fold	no change	no change	no change	Increase, 1.44-fold	no change	Increase, 1.41-fold	no change	Increase, 3.49-fold	81.82	Decrease,7.7-fold
**LC2**	Increase, 2.99-fold	Increase, 3.81-fold	Increase, 1.29-fold	Increase, 1.46-fold	Increase, 1.4-fold	Increase, 1.16-fold	no change	Increase, 1.27-fold	Increase, 1.93-fold	Increase, 2.95-fold	100	Decrease,8.06-fold
**LC3**	Increase, 4.82-fold	Increase, 4.16-fold	Increase, 1.22-fold	no change	Increase, 1.53-fold	Increase, 1.38-fold	no change	no change	Increase, 2.52-fold	Increase, 2.95-fold	100	Decrease,1.61-fold
**LC4**	Increase, 4.43-fold	Decrease, 3.85-fold	Increase, 1.27-fold	Decrease, 2.65-fold	Increase, 1.69-fold	Decrease, 1.66-fold	Increase, 1.17-fold	Decrease, 1.34-fold	Increase, 2.37-fold	Decrease, 4.43-fold	40.91	Increase,111.75-fold
**LC5**	Decrease, 2.2-fold	Decrease, 4.97-fold	no change	Decrease, 2.83-fold	no change	Decrease, 1.54-fold	Decrease, 1.23-fold	Decrease, 1.31-fold	Decrease, 2.19-fold	Decrease, 5.71-fold	45.45	Increase,117.99-fold
**LC6**	Decrease, 1.58-fold	Decrease, 4.21-fold	no change	no change	Decrease, 1.29-fold	Decrease, 1.28-fold	Decrease, 1.22-fold	Decrease, 1.26-fold	Decrease, 3.59-fold	Decrease, 5.78-fold	63.64	Increase,2.23-fold
**LC7**	no change	no change	no change	Decrease, 2-fold	no change	Decrease, 1.23-fold	no change	no change	no change	no change	86.36	Increase,12.78-fold
**Consortium**	Increase, 5.86-fold	Increase, 6.74-fold	Increase, 1.34-fold	Increase, 1.31-fold	Increase, 1.68-fold	Increase, 1.61-fold	Increase, 1.2-fold	Increase, 1.64-fold	Increase, 2.96-fold	Increase, 3.82-fold	100	Increase,5.25-fold

TableS3. Fold-changes of photosynthetic parameters in As-stressed plants upon endophyte treatment

Parameter	Significance	Fold change (+As+Endophyte/+As)
PI_{ABS}	Photosynthetic performance index	Increase, 10-fold
RC/ABS	Reaction center (RC) density per PSII antenna chlorophyll	Increase, 2.05-fold
DI_0/RC	Dissipation energy flux per PSII RC	Decrease, 3.95-fold
ET_0/RC	Maximum electron transport flux (further than Q_A^-) per PSII RC	Increase, 1.77-fold
TR_0/RC	Trapped (maximum) energy flux (leading to Q_A reduction) per RC	Decrease, 1.16-fold
ABS/RC	Light absorption flux per PSII RC	Decrease, 2.05-fold
Kp	Photochemical de-excitation rate constant in the excited antennae of energy fluxes for photochemistry	Increase, 2.85-fold
Kn	Non-photochemical de-excitation rate constant in the excited antennae for non-photochemistry	Decrease, 1.25-fold
Sum K	Sum of Kp and Kn	Increase, 1.67-fold
Sm	(Area)/($Fm-F_0$), representing energy necessary for the closure of all reaction centers	Increase, 3.68-fold
Sm/T (fmax)	Ratio representing th eaverage redox state of Q_A in the time span from 0 to T_{FM} (time required to reach Fm) and, concomitantly, the average fraction of open RCs during the time needed to complete their closure	Increase, 1.72-fold
N	Number of Q_A redox turnovers until Fm is reached	Increase, 3.33-fold
Vj	Relative variable fluorescence at time J	Decrease, 1.74-fold
dV/dt_0	Initial slope of the relative variable fluorescence in the fluorescence induction curve expressing the rate of accumulation of closed RCs.	Decrease, 2.02-fold

TableS4. Primers used for qRT-PCR.

Primer	Used for amplification of gene	Sequence (5'-3')	Purpose	PCR conditions (for cloning)
SnTIP2-1 Fwd	SnTIP2-1	GCTGAATTCATCTCCACACTCCTC	qRT-PCR	
SnTIP2-1 Rev	SnTIP2-1	TGGCAAACTGAATAGCTACGAGC	qRT-PCR	
SnATIP Fwd	SnATIP	CGTTGTCCGAAGGTGTATCAGTATG	qRT-PCR	
SnATIP Rev	SnATIP	CAATCACTCCCAAATCTCCCTTCT	qRT-PCR	
SnTIP2-2 Fwd	SnTIP2-2	TGTGGCTAGTGGAAACTTCGC	qRT-PCR	
SnTIP2-2 Rev	SnTIP2-2	GCTGGATAGAGGTGCATGTTCATG	qRT-PCR	
SnPIP1 Fwd	SnPIP1	CTGCTATCATCTACAACAACGATGC	qRT-PCR	
SnPIP1 Rev	SnPIP1	GACCTGTGGAATGGCATAGCTC	qRT-PCR	
SnMRP1 ExtFwd	SnMRP1	TATGCCGCCAAGATGTTGCATGATG	Cloning (Primary PCR)	98°C for 5min; 98°C for 30s, 60°C for 30s, 72°C for 35s, 40 cycles; 72°C for 5mins; 4°C for ∞.
SnMRP1 ExtRev	SnMRP1	CGGTATAATACCAAGAACCTTCCGT	Cloning (Primary PCR)	
SnMRP2 ExtFwd	SnMRP2	TCTAGTCTAAATGCAGCTAAAGGGA	Cloning (Primary PCR)	
SnMRP2 ExtRev	SnMRP2	AGGTATGATACTAAGAGCACTTCGA	Cloning (Primary PCR)	
SnMRP3 ExtFwd	SnMRP3	GCGACTTTCAGGATCCTCCAAGA	Cloning (Primary	

			PCR)	
SnMRP3 ExtRev	SnMRP3	CTCTTTGATTGCCTTC TGATCAG	Cloning (Primary PCR)	
SnMRP1 IntFwd	SnMRP1	GGGCCAGGTGTTTCA GCTTATCTCA	Cloning (Nested PCR) + qRT-PCR	98°C for 5min; 98°C for 30s, 65°C for 30s, 72°C for 25s, 40 cycles; 72°C for 5mins; 4°C for ∞.
SnMRP1 IntRev	SnMRP1	GGGCCTGTAACGTAA GACAACGTT	Cloning (Nested PCR)	
SnMRP2 IntFwd	SnMRP2	TAATACGTGTCTCAG CCAATTGTGG	Cloning (Nested PCR)	
SnMRP2 IntRev	SnMRP2	CAGGTCTGTAACGTA GGACAACATC	Cloning (Nested PCR) + qRT-PCR	
SnMRP3 IntFwd	SnMRP3	AGCAAGATGCAACCA TTGTGCTG	Cloning (Nested PCR) + qRT-PCR	
SnMRP3 IntRev	SnMRP3	ACCAATATCACATCA GCAGCTGGC	Cloning (Nested PCR)	
SnMRP1 RTRev	SnMRP1	GCTCTGATAATATAA ATAGGCTCCG	qRT-PCR	
SnMRP2 RTFwd	SnMRP2	ATAGATTTGCCATCA GAGGCTCA	qRT-PCR	
SnMRP3 RTRev	SnMRP3	CAACCTTTAATTGTAT GCCTGC	qRT-PCR	
ACT FRT1	Actin	CTTGCACCAAGCAGC ATGAA	qRT-PCR	
ACT RRT1	Actin	ACCGATCCAGACACT GTACTTCCTC	qRT-PCR	

Supplemental Figures

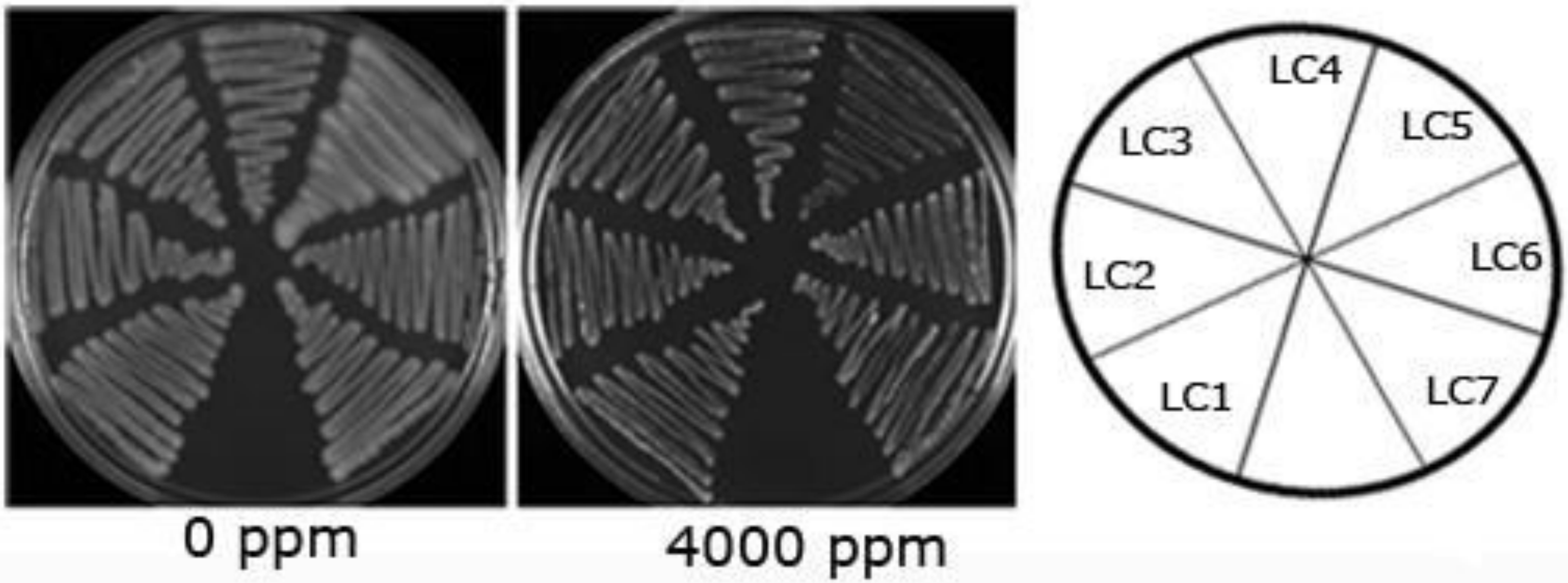

Fig.S1

Lantana camara endophytes were As tolerant.

L. camara endophytes were grown on TSB agar plates supplemented with different concentrations of As. Growth in the presence of 0 and 4000ppm As is shown. LC1.:*Enterobacter* sp. LC1, LC2.:*Kocuria* sp. LC2, LC3.:*Kocuria* sp. LC3, LC4.:*Enterobacter* sp. LC4, LC5.:*Kocuria* sp. LC5, LC6.:*Enterobacter* sp. LC6, LC7.:*Kosakonia* sp. LC7.

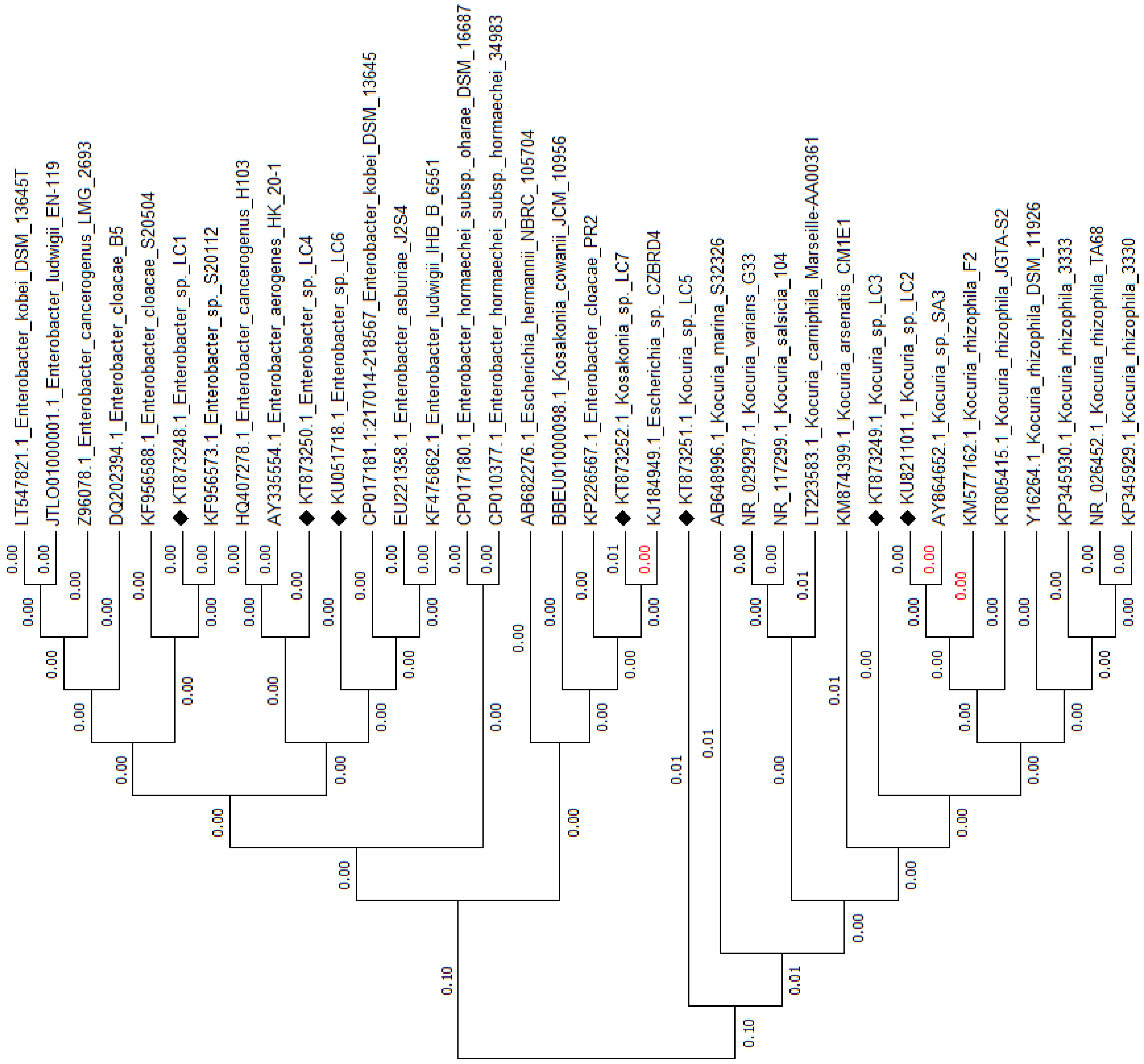
LT547821.1_Enterobacter_kobei_DSM_13645T
JTLO01000001.1_Enterobacter_ludwigii_EN-119
Z96078.1_Enterobacter_cancerogenus_LMG_2693
DQ202394.1_Enterobacter_cloacae_B5
KF956588.1_Enterobacter_cloacae_S20504
KT873248.1_Enterobacter_sp._LC1
KF956573.1_Enterobacter_sp._S20112
HQ407278.1_Enterobacter_cancerogenus_H103
AY335554.1_Enterobacter_aerogenes_HK_20-1
KT873250.1_Enterobacter_sp._LC4
KU051718.1_Enterobacter_sp._LC6
CP017181.1:217014-218567_Enterobacter_kobei_DSM_13645
EU221358.1_Enterobacter_asburiae_J2S4
KF475862.1_Enterobacter_ludwigii_IHB_B_6551
CP017180.1_Enterobacter_hormaechei_subsp._oharae_DSM_16687
CP010377.1_Enterobacter_hormaechei_subsp._hormaechei_34983
AB682276.1_Escherichia_hermannii_NBRC_105704
BBEU01000098.1_Kosakonia_cowanii_JCM_10956
KP226567.1_Enterobacter_cloacae_PR2
KT873252.1_Kosakonia_sp._LC7
KJ184949.1_Escherichia_sp._CZBRD4
KT873251.1_Kocuria_sp._LC5
AB648996.1_Kocuria_marina_S32326
NR_029297.1_Kocuria_varians_G33
NR_117299.1_Kocuria_salsicia_104
LT223583.1_Kocuria_carniphila_Marseille-AA00361
KM874399.1_Kocuria_arsenatis_CM1E1
KT873249.1_Kocuria_sp._LC3
KU821101.1_Kocuria_sp._LC2
AY864652.1_Kocuria_sp._SA3
KM577162.1_Kocuria_rhizophila_F2
KT805415.1_Kocuria_rhizophila_JGTA-S2
Y16264.1_Kocuria_rhizophila_DSM_11926
KP345930.1_Kocuria_rhizophila_3333
NR_026452.1_Kocuria_rhizophila_TA68
KP345929.1_Kocuria_rhizophila_3330

Fig. S2

Phylogenetic tree showing the relative distances of the 16S rRNA gene sequences of *L. camara* endophytes with their closest homologs. The tree is unrooted and prepared using Neighbour-Joining algorithm in Mega6. Our isolates have been indicated with black rhombus.

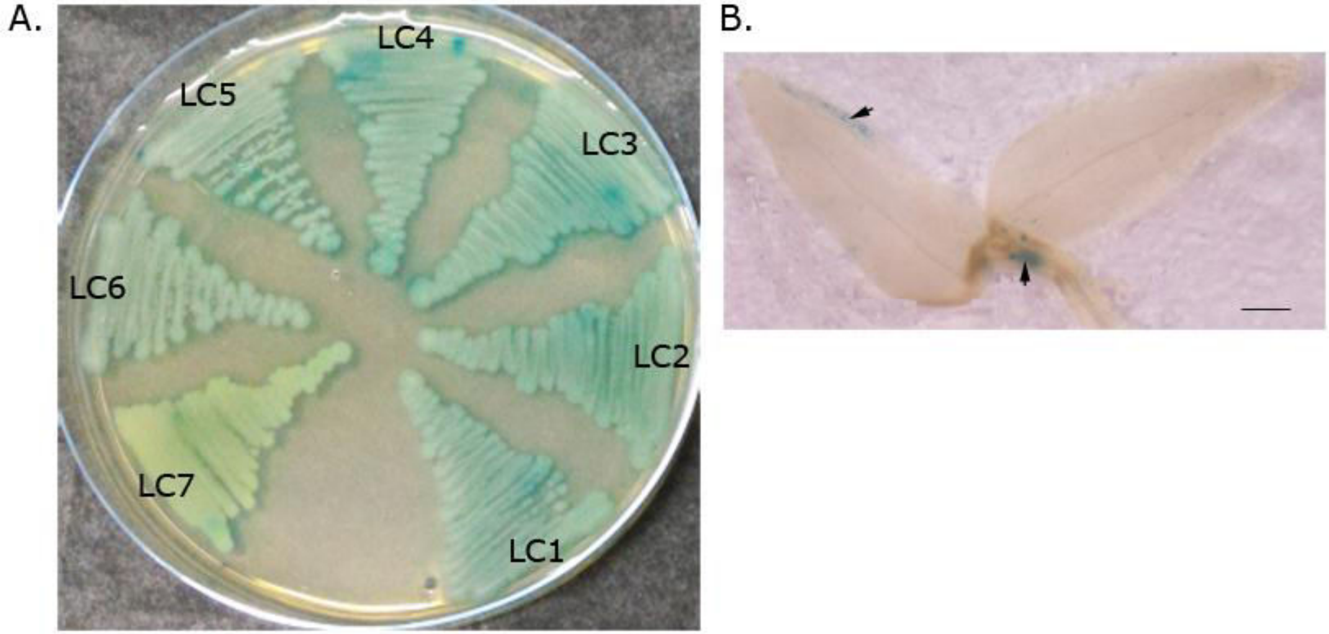

Fig. S3

L. camara endophytes could translocate into *S. nigrum* leaves.

(A) Endophytes labeled with pRJPaph-LacZYA were stained with 20µg/mL X-gal. LC1.:*Enterobacter* sp. LC1, LC2.:*Kocuria* sp. LC2, LC3.:*Kocuria* sp. LC3, LC4.:*Enterobacter* sp. LC4, LC5.:*Kocuria* sp. LC5, LC6.:*Enterobacter* sp. LC6, LC7.:*Kosakonia* sp. LC7 **(B)** One week old *S. nigrum* seedlings were infected with LacZ-labeled endophytes and stained with 100µg/mL X-gal. Stained regions of the leaf are shown with arrows indicating colonization. Establishment of *Enterobacter* sp. LC4 in a leaf of the plant was visualized under a Leica M60 stereo-microscope. Scale bar=1mm.

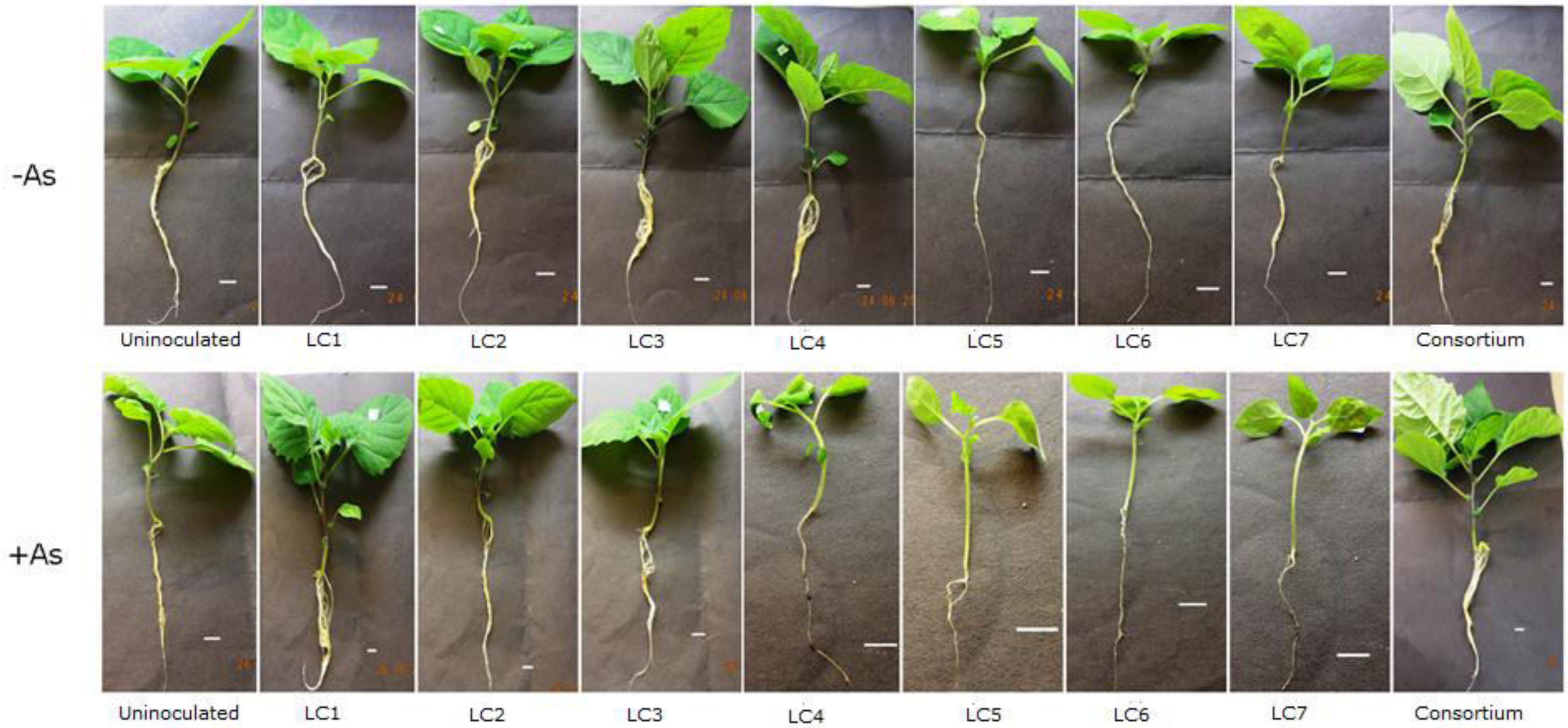

Fig. S4

L. camara endophytes have differential growth promoting effect on *S. nigrum*

Representative images of *S. nigrum* seedlings grown in presence of individual endophytes or consortium with or without As. LC1.:*Enterobacter* sp. LC1, LC2.:*Kocuria* sp. LC2, LC3.:*Kocuria* sp. LC3, LC4.:*Enterobacter* sp. LC4, LC5.:*Kocuria* sp. LC5, LC6.:*Enterobacter* sp. LC6, LC7.:*Kosakonia* sp. LC7. Scale bar=1cm.

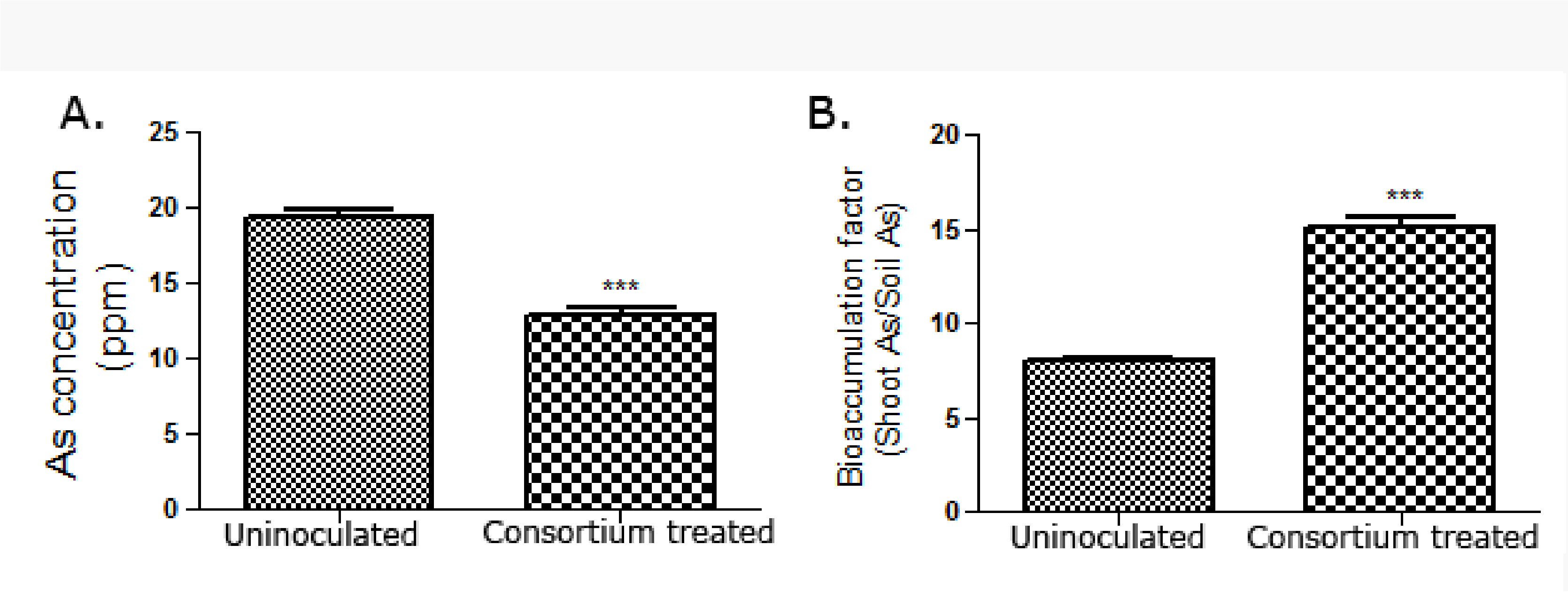

Fig. S5

L. camara endophytes increase As-bioaccumulation in *S. nigrum*.

(A) & (B) *S. nigrum* plants were grown in presence of As with or without the consortium and As concentration in rhizospheric soil (A) and bioaccumulation factor (B) were measured. ***P<0.0001 (unpaired two-tailed t-test; n=12).

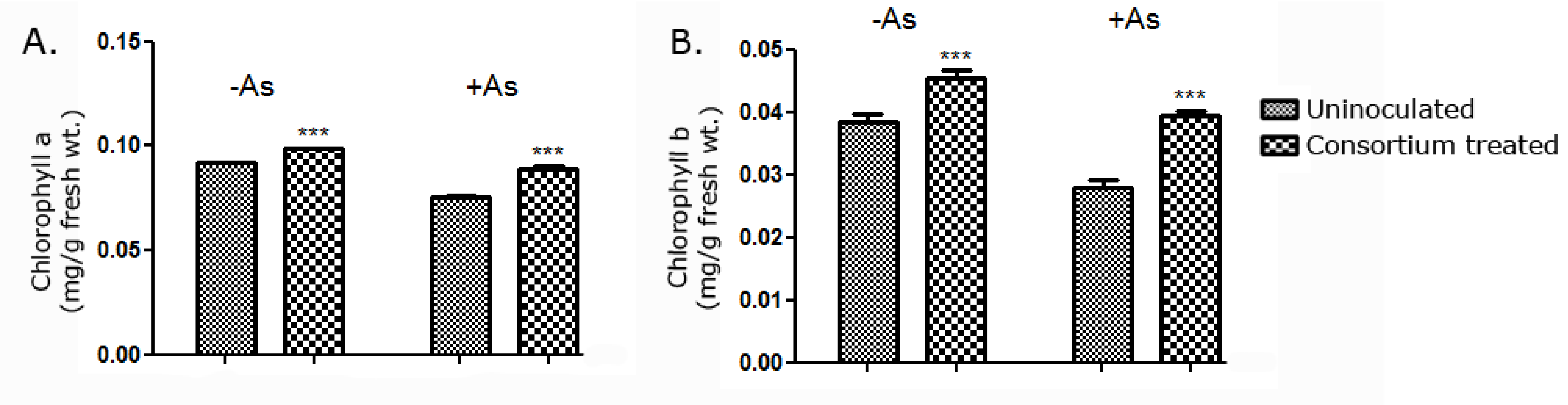

Fig.S6

L. camara endophytic consortium increase chlorophyll a and b content in *S. nigrum* leaves.

(A) & (B) Chlorophyll a (A) and b (B) content in plant leaves were compared between endophyte treated plants and untreated plants were grown in the presence of As. *** P<0.0001 (two-way ANOVA with Tukey's post-hoc test); n=9 (3 leaves/plant).

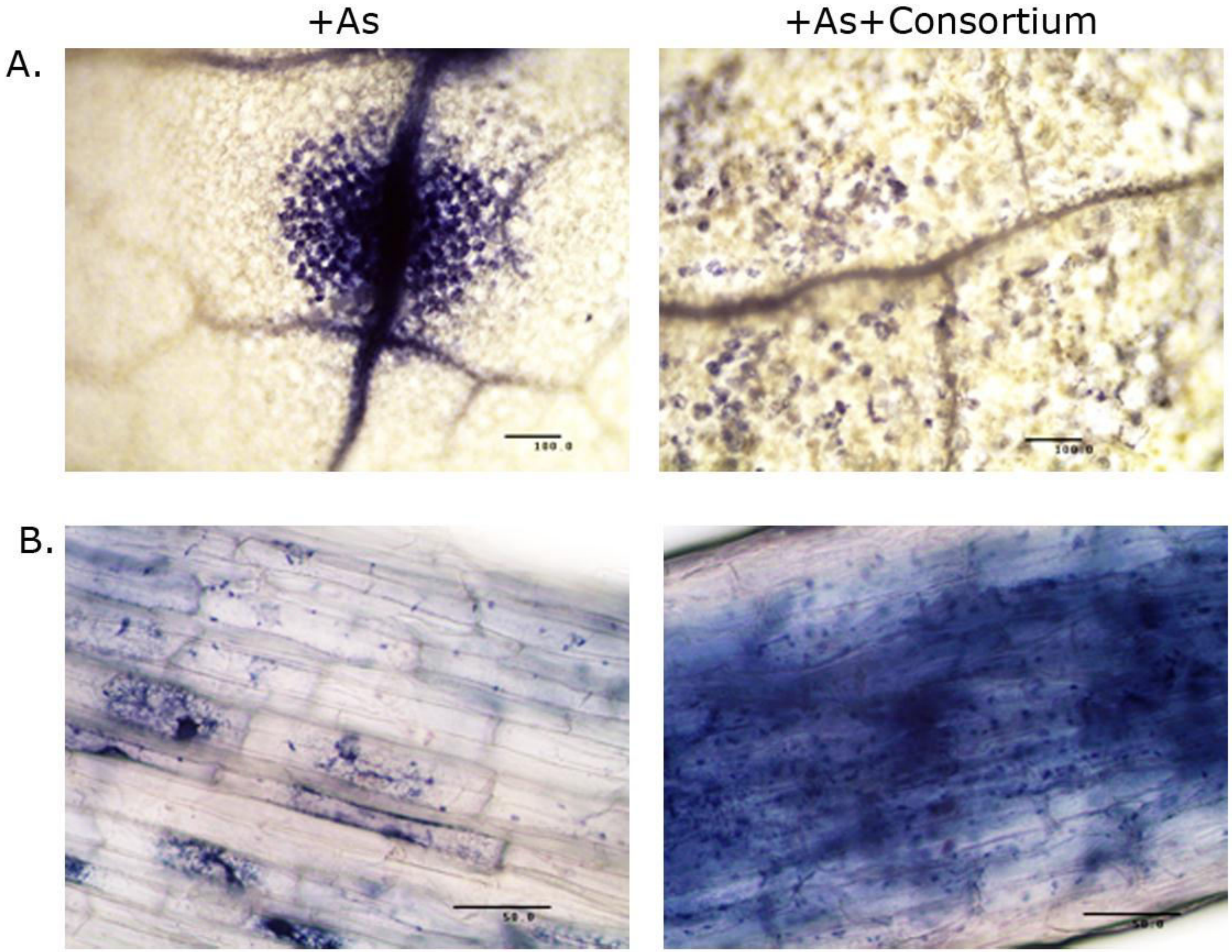

Fig. S7

L. camara endophytic consortium affected ROS distribution in *S. nigrum* in presence of As. Leaf and root of *S. nigrum* plants treated with or without endophytes grown in presence of As were stained with 0.05% NBT and visualized under a light microscope.

(A) NBT staining in leaf section. Scale bar=100µm.

(B) NBT staining in root section. Scale bar=50µm.

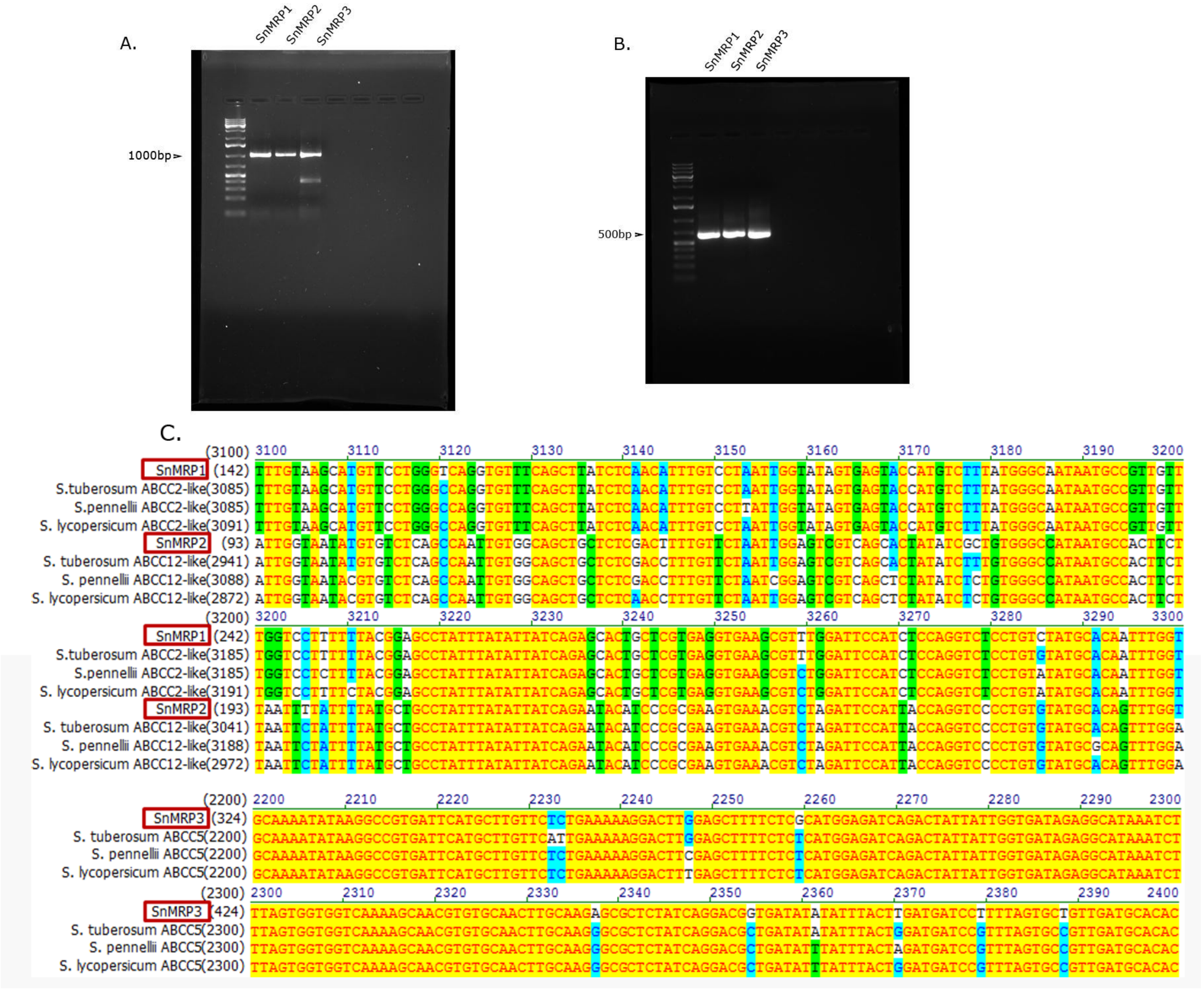
A.
SnMRP1
SnMRP2
SnMRP3
1000bp
B.
SnMRP1
SnMRP2
SnMRP3
500bp
C.
SnMRP1 (142)
S.tuberosum ABCC2-like(3085)
S.pennellii ABCC2-like(3085)
S. lycopersicum ABCC2-like(3091)
SnMRP2 (93)
S. tuberosum ABCC12-like(2941)
S. pennellii ABCC12-like(3088)
S. lycopersicum ABCC12-like(2872)
SnMRP1 (242)
S.tuberosum ABCC2-like(3185)
S.pennellii ABCC2-like(3185)
S. lycopersicum ABCC2-like(3191)
SnMRP2 (193)
S. tuberosum ABCC12-like(3041)
S. pennellii ABCC12-like(3188)
S. lycopersicum ABCC12-like(2972)
SnMRP3 (324)
S. tuberosum ABCC5(2200)
S. pennellii ABCC5(2200)
S. lycopersicum ABCC5(2200)
SnMRP3 (424)
S. tuberosum ABCC5(2300)
S. pennellii ABCC5(2300)
S. lycopersicum ABCC5(2300)

Fig. S8

Partial sequences of three ABCC transporters (SnMRP1, SnMRP2 and SnMRP3) were cloned from *S. nigrum*.

(A) & (B) Primary (A) and nested (B) PCR products of the SnMRPs amplified from *S. nigrum* cDNA.

(C) Alignment of three classes of SnMRP sequences with their homologs from *Solanum tuberosum, S. pennellii* and *S. lycopersicum.*
